# Immune response of DNA vaccinated-gilthead seabream (*Sparus aurata*) against LCDV-Sa infection: relevance of the inflammatory process

**DOI:** 10.3389/fimmu.2023.1209926

**Published:** 2023-06-06

**Authors:** Rocio Leiva-Rebollo, Juan Gémez-Mata, Dolores Castro, Juan J. Borrego, Alejandro M. Labella

**Affiliations:** Department of Microbiology, Faculty of Sciences, University of Malaga, Malaga, Spain

**Keywords:** LCDV-Sa, innate immune response, inflammation, DNA-vaccine, gilthead seabream, OpenArray^®^, DEGs

## Abstract

Lymphocystis disease is one of the main viral pathologies affecting cultured gilthead seabream (*Sparus aurata*) in the Mediterranean region. Recently, we have developed a DNA vaccine based on the major capsid protein (MCP) of the *Lymphocystis disease virus 3* (LCDV-Sa). The immune response triggered by either LCDV-Sa infection or vaccination have been previously studied and seem to be highly related to the modulation of the inflammatory and the IFN response. However, a comprehensive evaluation of immune-related gene expression in vaccinated fish after viral infection to identify immunogenes involved in vaccine-induced protection have not been carried out to date. The present study aimed to fulfill this objective by analyzing samples of head-kidney, spleen, intestine, and caudal fin from fish using an OpenArray^®^ platform containing targets related to the immune response of gilthead seabream. The results obtained showed an increase of deregulated genes in the hematopoietic organs between vaccinated and non-vaccinated fish. However, in the intestine and fin, the results showed the opposite trend. The global effect of fish vaccination was a significant decrease (*p*<0.05) of viral replication in groups of fish previously vaccinated, and the expression of the following immune genes related to viral recognition (*tlr9*), humoral and cellular response (*rag1* and *cd48*), inflammation (*csf1r*, *elam*, *il1β*, and *il6*), antiviral response (*isg15*, *mx1*, *mx2*, *mx3*), cell-mediated cytotoxicity (*nccrp1*), and apoptosis (*prf1*). The exclusive modulation of the immune response provoked by the vaccination seems to control the progression of the infection in the experimentally challenged gilthead seabream.

## 1 Introduction

Self-limited chronic Lymphocystis disease (LCD) is a well-known viral infection in fish that is characterized by the growth of small pearl-like nodules, with papilloma-like appearance, on the skin and fins of affected fish (1, 2). It has an incidence rate that can be as high as 70%, meaning it causes significant economic losses in the aquaculture sector due to the appearance of external lesions and the difficult commercialization of specimens with signs of the disease (3). This viral disease affects a wide variety of freshwater, brackish, and marine fish species, with *Lymphocystis disease virus 3* (LCDV-3, also named LCDV-Sa) being the main causative agent of LCD in gilthead seabream (*Sparus aurata*), and Senegalese sole (*Solea senegalensis*) in the Mediterranean and European South-Atlantic marine aquaculture (4–7). That said, recently LCDV belonging to genotype I, associated with LCD in Northern European countries, has also been reported to affect this fish species in Egypt (8). LCD lesions usually resolve one month after their appearance and are significantly influenced by water temperature. However, an asymptomatic carrier state is frequently detected in both gilthead seabream and Senegalese sole, with the detection of viral DNA and transcripts in a systemic distribution in fish tissues and organs. Asymptomatic infections are frequently detected in these fish species in fish farms, even when no sign of the disease or outbreaks are registered (4).

The genus *Lymphocystivirus* (Family *Iridoviridae*, subfamily *Alphairidovirinae*) has to date comprised four species: *Lymphocystis disease virus 1* (LCDV-1), isolated from European flounder (*Platichthys flesus*) and plaice (*Pleuronectes platessa*) in Europe; *Lymphocystis disease virus 2* (LCDV-2), isolated from Japanese flounder (*Paralichthys olivaceus*) in China; *Lymphocystis disease virus 3* (LCDV-3), isolated from gilthead seabream (*S. aurata*) in Spain; and *Lymphocystis disease virus 4* (LCDV-4), isolated from whitemouth croaker (*Micropogonias furnieri*) in Uruguay (9–13). These viruses have double-stranded DNA genomes, with icosahedral particles ranging from 130 to 300 nm in diameter, and nucleocytoplasmic replication (14).

The immune response of gilthead seabream against LCDV-3 involved in natural or experimental infections has been under-studied to date. In naturally infected fish, an impairment of the innate and adaptive immune response has been described, characterized by the presence of granular cells containing interleukin-1 beta (IL-1β) in perivascular sites and within capillaries, and also surrounding the lymphocysts, but with intense degranulation of acidophilic granulocytes or with no regulation of the transcript, diminished expression of antiviral genes *ifn*, *irf3*, and *mx*, a detriment of macrophages with down-regulation of *csf1r*, and also *mhcIIα*, *tcrα*, and *ighm* genes of antigen presentation cells (APC), and the main receptors of T and B cells, respectively (15, 16). Only a positive role in killing infected cells has been described for non-specific cytotoxic cells (NCCs) by the up-regulation of *nccrp1* (16). Regarding the immune response of gilthead seabream after experimental infection, a partial response of the type I interferon system in head-kidney and intestine and a lack of genes related to the inflammatory process in both organs have also been observed, results that agree with those obtained in naturally infected fish and that could favor the establishment of asymptomatic chronic infection. In addition, *nccrp1* was also up-regulated as it was described previously and postulated the NCCs as the main defensive mechanism of this fish species against this viral pathogen (17).

At present, there are no commercialized treatments or vaccines to prevent LCD, and the unique practices in hatcheries consist of controlling asymptomatic fish and/or food carriers, disinfectant procedures, and stocking density (18, 19). Recently, a plasmid DNA vaccine against LCDV-Sa has been developed by cloning the *mcp* (major capsid protein) gene into pcDNA3.1/NT-GFP-TOPO vector, and the protection conferred by the vaccine and the immune response induced in vaccinated fish was evaluated. The vaccine persists for at least 20 days with systemic distribution and *mcp* transcripts mostly detected in the head-kidney. In contrast to the results described during LCDV-Sa natural or experimental infections, the vaccine induced an inflammatory process by the overexpression of pro-inflammatory genes (*il1β*, *il6*, *casp1*, *ck3*, and *ck10*), and the down-regulation of the anti-inflammatory interleukin 10 (*il10*), also driving the production of specific neutralizing antibodies, conferring a possible protective state against LCDV-Sa. However, the type I interferon genes were not induced after the vaccination trials (20).

The relevance of the inflammatory response to control LCDV infection has been described in Japanese flounder and Senegalese sole as being critical to an effective innate and adaptive immune response to viral infections (21–23). It seems that in LCDV-infected gilthead seabreams, the opposite trend occurs, as inflammation was inhibited and early activation of *il10* was observed, which could be related to the development of persistent infection in this important cultured fish species (17, 24).

The present study aimed to evaluate the immune response of vaccinated-gilthead seabream juveniles after LCDV-Sa infection, analyzing the hematopoietic organs (head-kidney and spleen), the mucosal immunity (intestine), and the target organ/tissue of viral replication (fin) using an OpenArray^®^ platform consisting of 49 genes related to gilthead seabream immune response, with special emphasis on the inflammatory process as a potential marker of protection against LCD in this fish species.

## 2 Materials and methods

### 2.1 Fish maintenance

Gilthead seabream specimens (5-10 g weight) were obtained from a fish farm (Predomar SL, Almeria, Spain) and belonged to a single cohort. Fish were acclimated for two weeks before starting the experiment. The fish were maintained under natural photoperiod conditions and fed with a commercial pellet at a rate of 1% of the fish biomass administrated once per day. Water temperature and salinity conditions were 22 ± 1°C and 35–37 g L^-1^, respectively. During the acclimation stage, 10 fish were randomly analyzed by real-time PCR (qPCR) (25) to confirm a negative result for LCDV.

### 2.2 Vaccine preparation

The DNA vaccine used in the vaccination trial is based on the viral gene *mcp* (ORF LCDVSa062R, GenBank accession number KX643370.1) cloned into the eukaryotic expression vector pcDNA3.1/NT-GFP-TOPO (named pcDNA-MCP), following the manufacturer`s instructions (Invitrogen, Life Technologies Cop., Carlsbad, CA, USA). *Escherichia coli* One Shot TOP10 cells (Invitrogen) were transformed with pcDNA-MCP, and then the insert was confirmed by PCR and sequenced using primers and protocols previously described (20). For mock-vaccination trials, a re-ligated empty pcDNA3.1/NT-GFP-TOPO plasmid (pcDNA) was used.

*E. coli* containing pcDNA-MCP or pcDNA plasmids were conserved at -80 °C in LB broth, supplemented with ampicillin (100 µg mL^-1^), and glycerol (20%, vol/vol) as cryopreservant. The EndoFree Plasmid Mega Kit (Qiagen, Hilden, Germany) was used for plasmid purification, measuring its concentration by spectrophotometry using NanoDrop 1000 (Thermo Scientific, Life Technologies Co., Carlsbad, CA, USA). Purified plasmids were conserved at -20 °C until used.

### 2.3 Cell culture and viral isolate

SAF-1 cells were cultured in 25 cm^2^ flasks (Nunc) (Thermo Scientific) using growth medium (Leibovitz L-15 medium) (Gibco, Life Technologies Co., Carlsbad, CA, USA) supplemented with 1% penicillin-streptomycin (Sigma-Aldrich, Merck, Darmstadt, Germany), 2% L-glutamine (Sigma-Aldrich) and 10% fetal bovine serum (FBS) (Gibco).

The LCDV-Sa isolate used in this study was obtained from skin and fin lesions of diseased gilthead seabream specimens collected from a local farm (Southwestern Spain). Samples were homogenized (20% w/v) in an L-15 medium (Gibco). The cell suspension was sonicated at 40 W for 20 min and centrifuged (1000 x *g*, 5 min, 4 °C). The supernatant recovered was incubated with 10% penicillin-streptomycin overnight at 4 °C and stored at -80 °C. Viral titration was performed by end-point assays using SAF-1 cells grown on a 24-well plate. Cells were inoculated in triplicated with 200 µL per well of the appropriated viral dilution and incubated at 20 °C for 2 hours to ensure adsorption. The virus suspension was then replaced with 1 mL of maintenance medium (L-15 medium with 2% FBS and 1% penicillin-streptomycin). The cells were incubated at 20 °C and maintained for up to 14 days to observe CPE. The 50% cell culture infectious dose (TCID_50_) values were determined using the Reed and Muench method (26).

### 2.4 Experimental design

Gilthead seabream specimens (5 g mean weight) were separated into three experimental conditions and maintained in 100 L-capacity aquariums with independent water recirculation systems: 30 vaccinated fish (0.1 µg pcDNA-MCP/g fish dose), 30 mock-vaccinated specimens (same dose using pcDNA), and 60 fish were used as a control group (PBS, 100 µL). Animals were anesthetized with MS-222 (50 mg L^-1^) (Sigma-Aldrich) prior to the experiment being performed by intramuscular injection. Thirty days post-vaccination, the control group was divided into two groups, one injected with 100 µL of L-15 medium (negative control group), and the other one inoculated with the virus, establishing the non-vaccinated group. Vaccinated, mock-vaccinated, and non-vaccinated groups were inoculated with 100 µL of LCDV-Sa stock diluted in L-15 (10^6^ TCID_50_ per fish). Six fish per group were randomly selected at 24, 48, and 72 hours post-inoculation (pi). Prior to sampling, all fish were euthanized by MS-222 overdose (400 mg L^-1^). Samples from the caudal fin, intestine, head-kidney, and spleen were aseptically collected and stored at -80 °C until used.

All procedures were carried out under the Guidelines of the European Union Council (Directive 2010/63/EU) and the Spanish directive (RD 53/2013) for the protection of animals used in scientific experiments and authorized by the Spanish authorities for the regulation of animal care and experimentation (registration number 10-06-2016-102).

### 2.5 DNA-RNA extractions and cDNA synthesis

Samples of the selected organs were homogenized with Tri Reagent^®^ (Sigma-Aldrich), suspending the tissue (50-100 mg) in 1 mL and using the MM400 (Retsch, Haan, Germany) homogenizer. Afterward, 100 µL of 1-bromo-3-chloropropane (AppliChem, Darmstadt, Germany) was added and samples were centrifuged at 12.000 x *g* at 4 °C for 5 min. The aqueous phase was recovered and an equal volume of 75% ethanol was added. RNA extraction was carried out using the RNeasy Mini Kit (Qiagen) following the manufacturer’s instructions. RNA samples were quantified by spectrophotometry (NanoDrop 1000) ensuring their quality and integrity.

cDNA synthesis was carried out using MicroAmp Optical 96-well reaction plates (Applied Biosystems, Life Technologies Co., Carlsbad, CA, USA) and the High-Capacity cDNA Reverse Transcription Kit (Applied Biosystems). Each reaction contained 2 µg RNA, 2 µL of 10X RT Buffer, 2 µL of 10X RT Random Primers, 1 µL of 25X dNTPs, 1 µL of MultiScribe™ Reverse Transcriptase and 4 µL of RNase-free water. The synthesis profile was 10 min at 25 °C, 2 hours at 37 °C, 5 min at 85 °C, and during the final step, it was 4 °C.

DNA extractions were carried out using the E.Z.N.A. Tissue DNA Kit (Omega Bio-Tek Inc., Norcross, GA, USA) following the manufacturer’s instructions. DNA samples were suspended in DNase-free buffer, quantified spectrophotometrically, and stored at -20 °C until used.

### 2.6 LCDV-Sa detection and gene expression

Viral DNA quantification was carried out from caudal fin samples by qPCR in triplicate according to the procedure previously described (20), targeting a viral structural protein gene alternative to the *mcp* gene contained in the vaccine. The putative myristoylated membrane protein (*mmp*) gene (ORF LCDVSa074R, GenBank accession number KX643370.1) was used for qPCR assays. Amplification was performed using a 20-µL final volume reaction containing 12.5 µL of FastStart Essential DNA Green Master (Roche Diagnostics), 2 µL of each primer (10 pmol µL^-1^) (**Table 1**), and 200 ng of DNA. PCR amplifications were performed in a LighCycler^®^ 96 Instrument (Roche Diagnostics). The amplification profile was: initial denaturation at 95 °C for 10 min, followed by 40 cycles at 95 °C for 10 s, 60 °C for 10 s, and 72 °C for 10 s. Nonspecific amplification products were discarded by dissociation curve analyses following the thermal profile: 95 °C for 10 s, 65 °C for 60 s, and 97 °C for 1 s. LCDV-Sa DNA copy number was calculated by interpolation on a standard curve (20), and viral loads were expressed as *mmp* copies per microgram of DNA.

**Table 1:** Primers used for gene expression analysis by real-time PCR.

Abbreviation	Gene name	Sequence (5’-3’)	Amplicon size (bp)	Reference
*mmp*	Myristoylated membrane protein	F: TTGCCCCACTTCCTATTGTC	122	(20)
		R: CCGGTTTTTCAGACTTGGAA		

LCDV-Sa expression in caudal fin samples was quantified by real-time PCR from viral *mmp*. Viral RNA was extracted using the E.Z.N.A. total RNA kit, treated with RNase-free DNase I (Roche Diagnostics), and reverse-transcribed with the Transcription First Strand cDNA Synthesis Kit (Roche Diagnostics) following the manufacturer’s instructions and stored at -20 °C until used. Amplification was performed using a 20-µL final volume reaction according to the above-described conditions.

### 2.7 Gilthead seabream immune response after LCDV-Sa infection

To analyse the immune response in gilthead seabream after LCDV-Sa infection, qPCR reactions based on TaqMan™ probes were performed using an OpenArray^®^ platform (ThermoFisher Scientific). The array includes 49 target genes, which were included based on their important role in the fish immune response against viral infections and, in some of them, for their activity against LCDV-Sa infections (17). There were viral recognition-related genes (*tlr9, tlr5*, *cd209)*, inflammatory-related and cytokine genes (*c3*, *il1β, il6, il8, il10*, *tnfα, ck3, ck7, ck8, ck10, cox2, csf1r, ncf4, ccr3*, and *elam*), regulation of innate and adaptive immune response (*clec10a*, *tgfβ1*), antigen processing and presentation (*mhcIα*, *mhcIIα*, *iclp* and *mrc1*), type I IFN trigger genes and genes involved in IFN-1-dependent immune response (*irf1, irf3, irf9, ifn, pkr, isg15, mx1, mx2, mx3* and *ifi30)*, nonspecific cytotoxic cell receptor (*nccrp1)*, proteolysis process (*ctsb*), apoptotic process *(casp1, lgals1, perp, prf1)*, molecular stress response (*hsp70* and *hsp90*) and genes involved in humoral and cellular immune response (*tcrα, tcrβ, ighm, rag1, ilc, cd48* and *cd276*). Four genes have been selected as endogenous (*rps18, ub, actβ*, and *ef1α*). Primers and probes were designed using the Custom TaqMan™ Assay Design Tool with the option TaqMan™ Gene Expression Assays (Life Technologies). Selected transcripts, assay ID, assay sequences, primers, and TaqMan™ probes (Reporter dye FAM) and 3’ non-fluorescent quencher (NFQ) are indicated in **Supplementary Table S1**.

Quantitative PCRs were performed in the OpenArray^®^ system QuantStudio 12K Flex Real-Time PCR System (Applied Biosystems), sited in the Research Central Service of the University of Cordoba (Spain), using the TaqMan™ OpenArray^®^ Real-Time PCR Master Mix kit (Applied Biosystems). Samples were loaded in triplicate into OpenArray^®^ plates. For gene expression analysis, Ct values were obtained using the Thermo Fisher Connect™ (ThermoFisher Scientific) online application, and the Relative Quantification (RQ) software. The setup was adjusted with options Benjamini-Hochberg deactivated, maximum Ct was set up at 28, AMP score was activated and HIGHSD was changed to 0.25. Fold change (FC) values were obtained by the 2^− ΔΔCt^ method (27). Values were normalized with endogenous gene *rps18*, which showed a more stable expression by OpenArray^®^, according to their score values, which were obtained using the Applied Biosystems™ Relative Quantitation Analysis Module (ThermoFisher cloud dashboard), and indicate how the Ct values for a specific endogenous gene varied between samples compared to the other genes used as endogenous. Samples from the control group (non-infected) were used as the calibrator. To identify the differentially expressed genes (DEGs) involved in gilthead seabream immune response against LCDV-Sa infection, genes with log_2_ fold change < -0.5 (down-regulated) or > 0.5 (up-regulated) and *p*<0.05 were considered DEGs. A cluster analysis of the samples, based on the log_2_ fold change of the host genes, was conducted using the Expression Heat Map option on the web server Heatmap-per (http://www2.heatmapper.ca/) (28) with Euclidean as distance measurement method, and complete linkage as a clustering method. In addition, the expressed genes were also clustered using the same parameters. The Venn diagram method was used for the comparative analysis of DEG datasets obtained in each experimental group and the timepoint analyzed after the infection with the virus (29).

### 2.8 Statistical analysis

The qPCR data were log-transformed to get normality and homogeneity of variance, and the normality of the data was analyzed using a Shapiro-Wilk test. To determine significant differences in viral load or gene expression levels between groups and/or time points, a one-way ANOVA followed by Fisher’s LSD test was used. Differences were considered significant when *p*<0.05. The statistical tests were performed using GraphPad Prism version 8.0.0 for Windows, GraphPad Software, Sand Diego, California USA, www.graphpad.com.

## 3 Results

### 3.1 Viral load and gene expression in gilthead seabream

**Figure 1:**
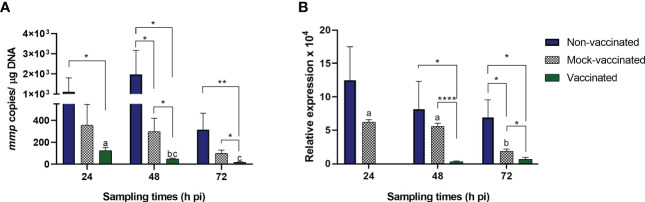
**(A)** Viral load (mmp copies/µg DNA) and **(B)** *mmp* gene relative expression in LCDV-Sa-infected gilthead seabreams analyzed by qPCR. Data are expressed as mean ± SEM (n = 6). Asterisks denote significant differences between experimental groups at a timepoint (*p<0.05, **p<0.01, ****p<0.0001). Letters establish significant differences through timepoint analyzed in a specific group.

To study the course of LCDV-Sa infection in gilthead seabream, viral load and *mmp* gene expression in caudal fin samples were analyzed by qPCR at 24, 48, and 72 hours pi for vaccinated, mock-vaccinated, and non-vaccinated animals inoculated with the virus by intramuscular injection (**Figure 1**). No signs of LCD were observed in any group at any time analyzed. LCDV-Sa was detected in all samples and timepoints in the infected groups, whereas no amplification was registered in the control group (L-15). Viral load in caudal fin samples at different times pi is shown in **Figure 1A**. Through the experiment, significant differences in viral load were observed between the non-vaccinated and vaccinated fish at all timepoints (1.1 ± 0.69 x 10^3^, 1.97 ± 1.20 x 10^3^, 3.15 ± 1.49 x 10^2^, and 1.24 ± 0.26 x 10^2^, 4.84 ± 0.65 x 10^1^, 1.76 ± 0.52 x 10^1^ copies of viral DNA µg^-1^ of tissue, respectively) (*p*<0.05 at 24- and 48-hours pi, *p*<0.01 at 72 hours pi). Regarding mock-vaccinated animals, significant differences were observed with the vaccinated animals at 48- and 72-hours pi (*p*<0.05) (2.97 ± 1.22 x 10^2^ and 9.83 ± 2.97 x 10^1^ copies of viral DNA µg^-1^ of tissue, respectively), and with the non-vaccinated animals only at 48 hours pi (*p*<0.05) where the highest viral load was detected. In all cases, the viral load in the vaccinated group had the lowest values at any timepoint analyzed, and also significant differences were observed within the group from 24 to 48 hours pi (*p*<0.0113) and 24 to 72 hours pi (*p*<0.0008), with a constant decrease in the viral load. The *mmp* gene expression in caudal fin samples is shown in **Figure 1B**. Significant differences were detected between vaccinated fish and the other groups at 48 and 72 hours pi (*p*<0.0001 and *p*<0.05 for mock-vaccinated, and *p*<0.05 for non-vaccinated animals, respectively). No expression of the *mmp* gene was detected in the vaccinated animals at 24 hours pi.

### 3.2 Immune response of gilthead seabream infected with LCDV-Sa

**Figure 2:**
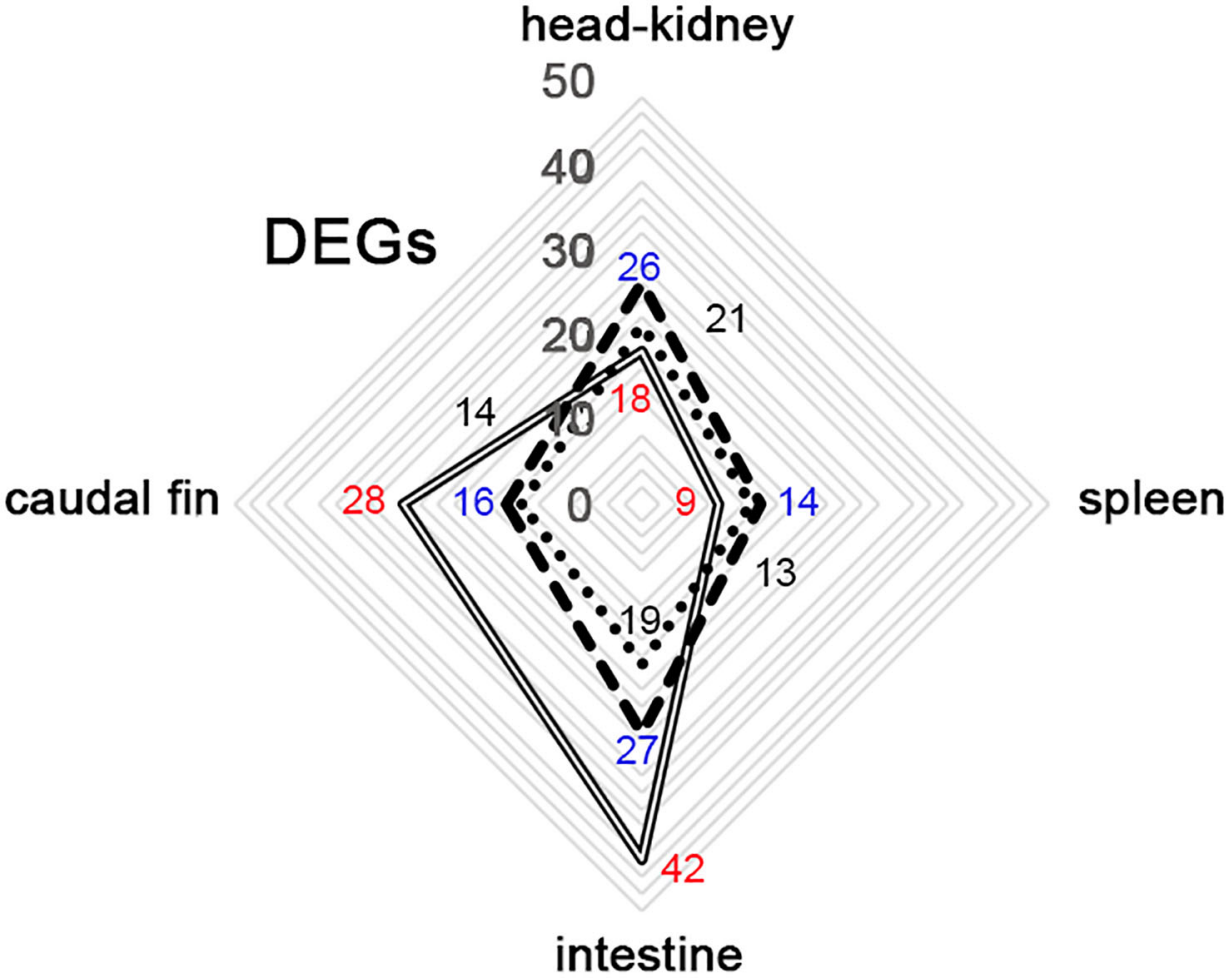
Distribution of differential expressed genes (DEGs) between organs from gilthead seabream infected with LCDV-Sa in non-vaccinated (full-line), vaccinated (segmented-line), and mock-vaccinated (dotted-line) animals through the whole experiment. Number of DEGs detected in the different experimental groups: in blue (vaccinated), black (Mock-vaccinated), and red (non-vaccinated).

In the present study, we evaluated the immune response in gilthead seabream juveniles after infection with LCDV-Sa using an OpenArray^®^ carrying 49 different assays related to the immune system. Only 4 out of the 49 genes were not differentially expressed during the experiment in any group; those genes were involved in the inflammatory process (*c3* and *ck7*), the interferon response (*ifn*), and antigen processing and presentation (*mhcIα*). Differentially expressed genes (DEGs) were analyzed in 4 different organs (head-kidney, spleen, intestine, and caudal fin) at 24, 48, and 72 hours pi. Regarding vaccinated animals, the number of DEGs detected was higher in the head-kidney and intestine (26 and 27, respectively) than in the spleen and caudal fin (14 and 16, respectively). A similar profile was detected in mock-vaccinated animals being 21 and 19 DEGs detected in the head-kidney and intestine, and 13 and 14 in the spleen and caudal fin. However, in the non-vaccinated group, a different profile of DEGs was detected, with the intestine and caudal fin being the organs where the highest number of DEGs was observed (42 and 28, respectively). Moreover, in the hematopoietic organs, the number of DEGs detected was lower than that observed in vaccinated and mock-vaccinated animals (18 and 9, for head-kidney and spleen) (**Figure 2**).

Attending to the type of gene regulation, down-regulation of genes was the major event in the head-kidney, intestine, and caudal fin for all the experimental groups analyzed (66.7, 66.7, and 78.6%, respectively in non-vaccinated animals) (57.7, 77.8, and 68.7%, respectively in vaccinated animals) (52.4, 94.7, and 64.3%, respectively in mock-vaccinated animals), with the exception of spleens where a different trend was detected with major up-regulation of genes (88.9% in non-vaccinated animals) (85.7% in vaccinated animals) (92.3% in mock-vaccinated animals) (**Table 2**). Regarding the tendency of gene regulation between experimental groups, organs, and timepoints analyzed, there was a change of regulation observed exclusively in vaccinated animals compared to the other groups, registered in the intestine, head-kidney, and caudal fin at 24, 48- and 72-hours pi, respectively, shifting from down- to up-regulation of DEGs. In this experimental group, the opposite trend (up- to down-regulation) was also denoted in the caudal fin and intestine at 24 and 72 hours pi with respect to non-vaccinated animals, but in this case, these profiles of regulation were also identified in the mock-vaccinated group (**Table 2**). Specific genes that were differentially expressed in the organs analyzed are discussed in detail below. Fold change (FC) values will be shown as Log_2_ FC to clearly explain the type of gene regulation (negative values for down-regulation and positive values for up-regulation).

**Table 2:** Differentially expressed genes (DEGs) detected in gilthead seabream post-infection (pi) with LCDV-Sa.

Experimental group	DEGs	Head-kidney			Spleen			Intestine			Caudal-fin		
		24h	48h	72h	24h	48h	72h	24h	48h	72h	24h	48h	72h
Non-vaccinated	Up-regulated	3	0	3	5	1	2	5	0	9	6	0	0
	Down-regulated	9	3	0	1	0	0	9	15	4	1	14	7
Vaccinated	Up-regulated	1	5	5	4	3	5	4	1	1	0	1	4
	Down-regulated	15	0	0	2	0	0	3	15	3	6	4	1
Mock-vaccinated	Up-regulated	2	0	8	0	3	9	1	0	0	2	3	0
	Down-regulated	8	2	1	1	0	0	2	12	4	3	5	1

#### 3.2.1 DEGs in head-kidney

**Figure 3:**
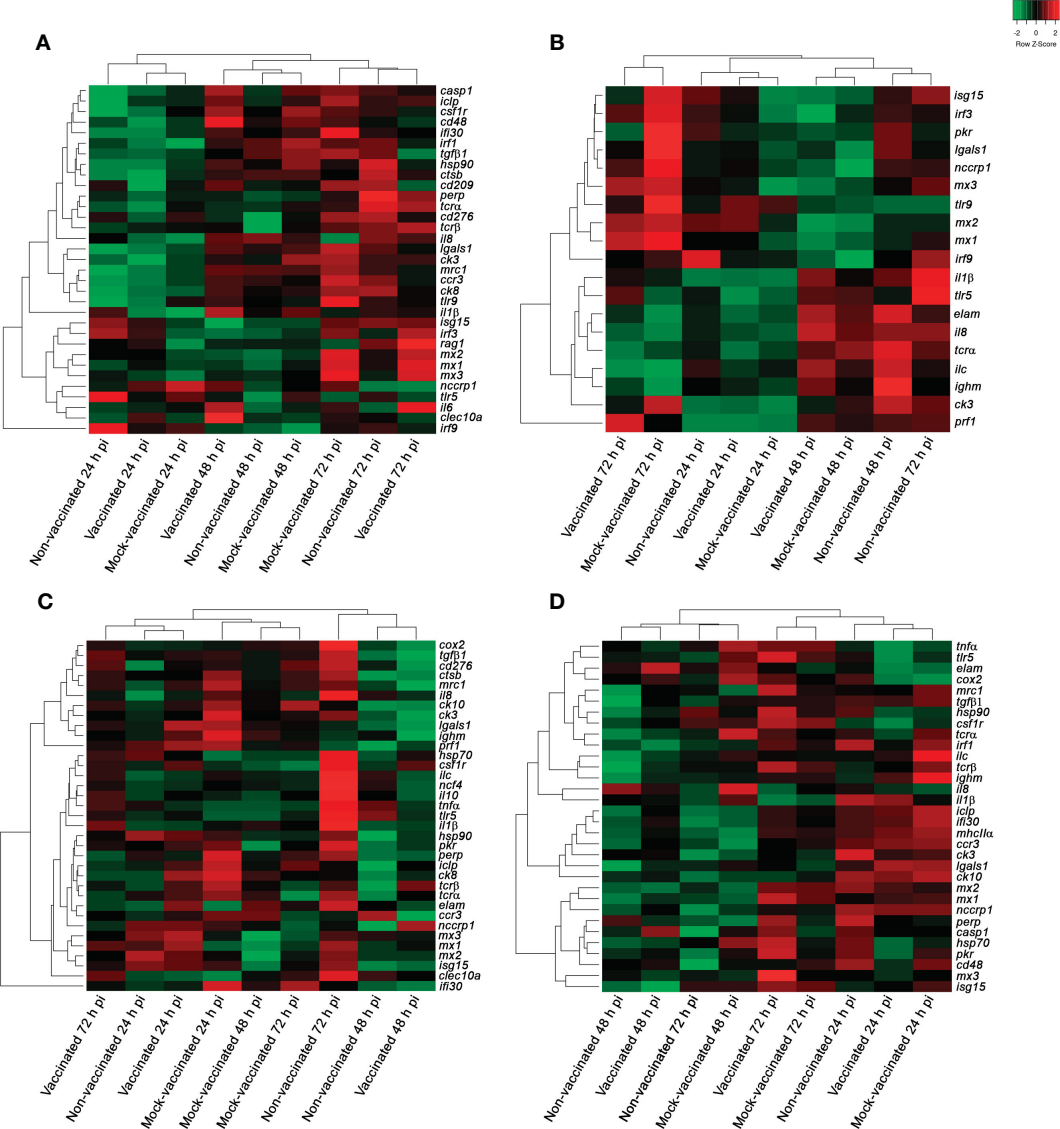
Hierarchical clustering analysis of samples from non-vaccinated, vaccinated, and mock-vaccinated LCDV-Sa infected gilthead seabreams at 24, 48, and 72 h pi. **(A)** Head-kidney, **(B)** spleen, **(C)** intestine, and **(D)** caudal fin samples. Green and red colors indicate down- and up-regulation of genes according to the scale shown [Log_2_ (FC)].

In the head-kidney samples of infected gilthead seabreams, 33 DEGs were detected between experimental groups and timepoints analyzed (**Figure 3A**). Regarding samples, the different experimental groups clustered to the timepoint of analysis, showing a general change of gene regulation from 24 h to 48- and 72-hours pi (down- to up-regulation of genes). The interferon regulatory factor 9 (*irf9*) gene had a strong modulation in this organ (FC of -2.62 in mock-vaccinated fish at 48 hours pi to 2.92 in non-vaccinated animals at 24 hours pi) (**Supplementary Tables S2**-**4**).

In non-vaccinated animals at 24 hours pi 9 DEGs were detected, all down-regulated and related to different processes of the immune response as viral recognition (*tlr9* with FC of -1.60), regulation of innate and adaptive immune response (*tgfβ1* with FC of -0.71), the type I interferon system (*irf1* with FC of -0.58), inflammation (*ck3* and *ck8*, with FC values of -1.60 and -1.18, respectively), antigen processing and presentation (*iclp* with FC of -0.86), apoptosis (*casp1* and *lgals1*, with FC values of -0.60 and -2.25, respectively), and molecular stress response (*hsp90* with FC of -0.51). On the other hand, only 3 DEGs were up-regulated and related to viral recognition (*tlr5* with FC of 1.05) and the interferon response (*irf9* and *mx2*, with FC values of 2.92 and 0.58, respectively). At 48 hours pi, the down-regulation of genes continued being predominant, with the detection of 3 DEGs related to inflammation (*ck3* with FC of -0.985) and cell-mediated response (*tcrβ* and *cd276* with FC values of -1.32 and -0.68, respectively). When infection progressed, up-regulation of the following genes were detected exclusively in *isg15* (FC of 0.93), *rag1* (FC of 1.21), and *perp* (FC of 0.6), indicating a change of tendency and slight promotion of antiviral response (**Supplementary Table S2**).

**Figure 4:**
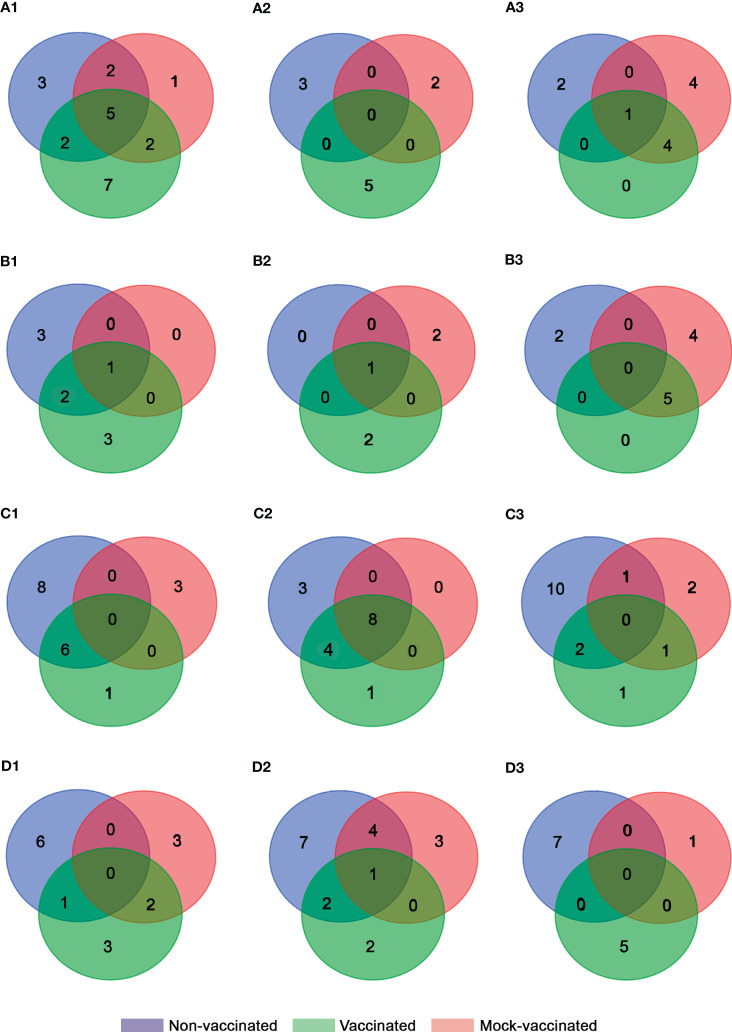
Venn diagram analysis of DEGs obtained for each experimental group from **(A)** head-kidney, **(B)** spleen, **(C)** intestine, and **(D)** caudal fin samples at (1) 24 (2), 48, and (3) 72 h pi.

Regarding vaccinated animals, at 24 hours pi a major down-regulation of genes was also recorded related to viral recognition (*tlr9* and *cd209* with FC values of -1.32 and -0.97, respectively), the regulation of innate and adaptive immune response (*tgfβ1* with FC of -0.74), type I interferon system (*irf1* and *ifi30* with FC of -0.74 and -0.92, respectively), inflammatory process (*ck3*, *ccr3*, *ck8* and *csf1r* with FC of -2.12, -1.15, -0.94, and -0.6, respectively), antigen processing and presentation (*mrc1* with FC of -1.18), cellular-mediated response (*tcrα* and *cd48* with FC values of -0.54 and -0.86, respectively), proteolysis and apoptosis (*ctsb* and *lgals1* with FC values of -0.79 and -1.89, respectively), and stress response (*hsp90* with FC of -0.51). However, the recombination-activating gene 1 (*rag1* with FC of 0.93) was the only gene up-regulated at this timepoint indicating an early cell-mediated response in comparison with the non-vaccinated animals (**Supplementary Table S3**). At this timepoint, 7 DEGs were exclusively detected in this group (*cd209*, *ifi30*, *csf1r*, *tcrα*, *cd48*, *ctsb*, and *rag1*). At 48 hours pi, a clear change of expression profile was recorded with up-regulation of genes related to the regulation of the innate and adaptive immune response (*clec10a* with FC of 1.48), inflammation (*il1β* and *il6* with FC values of 2.05 and 0.86, respectively), and cell-mediated response (*cd48* and *nccrp1* with FC values of 0.71 and 1.12, respectively) (**Supplementary Table S3**). Comparatively, the non-vaccinated animals showed a stronger antiviral response mediated by the interferon-related genes at 72 hours pi with the detection of *isg15*, *irf3*, *mx1*, *mx2*, and *mx3*, all of which were up-regulated with FC values of 0.86, 0.72, 1.92, 1.89, and 2.01, respectively (**Figure 4A1-3**, **Table 3**, **Supplementary Tables S2**-**3**).

**Table 3:** DEGs commonly or exclusively detected between non-vaccinated, vaccinated, and mock-vaccinated fish after infection with LCDV-Sa.

Shared DEGs	24 h pi	48 h pi	72 h pi
Head-kidney			
Non-vaccinated, vaccinated, mock-vaccinated	*tgfβ1, irf1, ck3, ck8, lgals1*	ND^a^	*isg15*
Non-vaccinated, vaccinated	*tlr9, hsp90*	ND	ND
Non-vaccinated, mock-vaccinated	*tlr5, irf9*	ND	ND
Vaccinated, mock-vaccinated	*ccr3, mrc1*	ND	*irf3, mx1, mx2, mx3*
Non-vaccinated	*mx2, iclp, casp1*	*ck3, tcrβ, cd276*	*rag1, perp*
Vaccinated	*cd209, ifi30, csf1r, tcrα, rag1, cd48, ctsb*	*clec10a, il1β, il6, cd48, nccrp1*	ND
Mock-vaccinated	*il8*	*tlr9, irf9*	*ifi30, il8, nccrp1, lgals1*
Spleen			
Non-vaccinated, vaccinated, mock-vaccinated	*ck3*	*ighm*	ND
Non-vaccinated, vaccinated	*mx1, mx2*	ND	ND
Vaccinated, mock-vaccinated	ND	ND	*mx1, mx2, mx3, nccrp1, prf1*
Non-vaccinated	*irf9, pkr, isg15*	ND	*tlr5, il1β*
Vaccinated	*tlr9, elam, nccrp1*	*elam, ilc*	ND
Mock-vaccinated	ND	*il8, tcrα*	*tlr9, irf3, pkr, lgals1*
Intestine			
Non-vaccinated, vaccinated, mock-vaccinated	ND	*tgfβ1, ck3, ck10, mrc1, cd276, ctsb, lgals1, hsp90*	ND
Non-vaccinated, vaccinated	*isg15, mx1, ifi30, il8, ck3, nccrp1*	*ifi30, ck8, nccrp1, prf1*	*ifi30, il1β*,
Non-vaccinated, mock-vaccinated	ND	ND	*lgals1*
Vaccinated, mock-vaccinated	ND	ND	*il8*
Non-vaccinated	*tgfβ1, mx2, mx3, ccr3, ck10, mrc1, cd276, lgals1*	*iclp, tcrα, tcrβ*,	*tlr5, clec10a, isg15, il10, ck10, csf1r, ncf4, ilc, prf1, hsp70*
Vaccinated	*csf1r*	*perp*	*ck3*
Mock-vaccinated	*tnfα, elam, perp*	ND	*ccr3, ighm*
Caudal fin			
Non-vaccinated, vaccinated, mock-vaccinated	ND	*mhcIIα*	ND
Non-vaccinated, vaccinated	*tcrβ*,	*tcrβ, ilc*	ND
Non-vaccinated, mock-vaccinated	ND	*ifi30, ccr3, ck10, iclp*	ND
Vaccinated, mock-vaccinated	*cox2, elam*,	ND	ND
Non-vaccinated	*pkr, il1β, cd48, nccrp1, casp1, perp*	*tgfβ1, csf1r, mrc1, tcrα, ighm, lgals1, hsp90*	*tlr5, ifi30, ck3, ccr3, ck10, mhcIIα, iclp*
Vaccinated	*tnfα, csf1r, tcrα*	*irf1, casp1*	*isg15, mx1, mx2, mx3, il8*
Mock-vaccinated	*tlr5, ccr3, ck10*,	*isg15, il8, hsp70*	*ilc*

a ND, non-detected.

Regarding the mock-vaccinated group, DEGs were mainly shared with the other experimental groups at 24- and 72-hours pi. However, the exclusive down-regulation of genes related to viral recognition (*tlr9* with FC of -0.775 at 48 hours pi), inflammation (*il8* with FC values of -1.25 and -1.18 at 24- and 72-hours pi), and regulation of interferon response (*irf9* with FC of -2.62 at 48 hours pi) was detected. In addition, up-regulation of genes related to interferon induced proteins (*ifi30* with FC of 1.07), non-specific cell-mediated response (*nccrp1* with FC of 1.12), and apoptosis (*lgals1* with FC of 0.91) were detected at 72 hours pi (**Figure 4A1-3**, **Table 3**, **Supplementary Tables S-4**).

#### 3.2.2 DEGs in spleen

In spleen samples, 19 DEGs were detected between experimental groups and the timepoints analyzed (**Figure 3B**). In this organ, the lowest modulation of differential gene expression was detected in the entire experiment. Regarding samples, three clusters of gene expression profiles were obtained, where all the samples of the different experimental groups clustered together at 24 hours pi. However, a clear distinction between groups was obtained at 48- and 72-hours pi, establishing a separate cluster for vaccinated and mock-vaccinated fish at the last timepoint of analysis (**Figure 3B**). The chemokine 3 (*ck3*) gene registered the lowest fold change value (-1.24 in mock-vaccinated fish at 24 hours pi) in this organ. On the contrary, the perforin 1 (*prf1*) gene had strong up-regulation in the vaccinated group (6.28 at 72 hours pi) (**Supplementary Tables S5**-**7**).

In non-vaccinated animals, a higher antiviral interferon-related response was only detected early on in the infection (24 hours pi), with the up-regulation of *irf9*, *pkr*, *isg15*, *mx1*, and *mx2* (FC values of 2.15, 0.81, 0.89, 1.29, and 1.74, respectively) in comparison to vaccinated and mock-vaccinated groups. In vaccinated fish, only *mx1* and *mx2* were up-regulated at 24 hours pi (FC values of 1.18 and 1.82, respectively). However, later on during the infection (72 hours pi) the three Mx were detected (FC values of 2.62, 2.08, and 1.65, respectively), indicating sustained interferon response during the infection. In contrast, in mock-vaccinated animals, the interferon pathway was only up-regulated at 72 hours pi but with a more complete gene profile (*irf3*, *pkr*, *mx1*, *mx2*, and *mx3*, with FC values of 1.14, 1.55, 3.07, 2.38, and 1.83) (**Figure B1-3**, **Table 3**, **Supplementary Tables S5**-**7**).

Regarding inflammation, early down-regulation of genes was observed in all the groups, with chemokine 3 (*ck3*) most commonly detected (**Figure 4B1**). Only in vaccinated animals, the endothelial leukocyte adhesion molecule (*elam*) was initially down-regulated (FC of -0.85 at 24 hours pi). However, at 48 hours pi the modulation of this gene changed and was up-regulated in these fish (FC of 1.71), promoting inflammation. In mock-vaccinated fish, the promotion of inflammation occurred at the same timepoint as in vaccinated fish with the up-regulation of the interleukin 8 (*il8*) gene (FC of 0.93). This is contrary to the pro-inflammatory response in non-vaccinated fish, which was not detected until 72 hours pi mediated by the up-regulation of interleukin 1 subunit beta (*il1β*) gene (FC of 2.06) (**Table 3**, **Supplementary Tables S5**-**7**).

In addition, in non-vaccinated animals, genes related to the humoral response (*ighm* with FC of 1.06) and viral recognition (*tlr5* with FC of 1.94) were detected at 48- and 72-hours pi, respectively. Interestingly, in vaccinated and mock-vaccinated animals, the toll-like receptor 9 (*tlr9*) was the nucleic acid sensor up-regulated, with an earlier modulation in vaccinated fish (FC of 1.03 at 24 hours pi) compared to the mock group (FC of 1.78 at 72 hours pi). Non-specific cell mediated response, humoral immune markers, and apoptosis were also promoted in these fish (vaccinated and mock), with the up-regulation gene profile of *ighm* and *ilc* (FC values of 1.34 and 1.25 at 48 hours pi), *nccrp1* (FC values of 0.7 and 0.87 at 24 and 72 hours pi, respectively), and strong modulation of *prf1* (FC of 6.28 at 72 hours pi) in vaccinated fish, and *tcrα* and *ighm* (FC values of 0.86 and 0.94 at 48 hours pi), *nccrp1* (FC of 1.42 at 72 hours pi), *lgals1* and *prf1* (FC values of 1.4 and 2.38 at 72 hours pi, respectively) in the mock animals (**Table 3**, **Supplementary Tables S5**-**7**).

#### 3.2.3 DEGs in the intestine

In intestine samples 35 DEGs were detected between experimental groups and the timepoints analyzed (**Figure 3C**). In this organ, samples of the different experimental groups constituted 3 clusters, where all the samples of the mock-vaccinated fish clustered together at the three timepoints analyzed. Although gene expression profiles of samples from vaccinated and non-vaccinated fish were more similar, establishing two clusters at 24- and 48-hours pi, a clear distinction of gene profiles between them was observed at 72 hours pi (**Figure 3C**). The genes with strong modulation in this organ were the interferon-gamma-inducible protein 30 (*ifi30*) gene (-5.44 at 48 h pi in vaccinated fish) and interleukine-1β (*il1β*) (2.41 at 72 hours pi in non-vaccinated fish) (**Supplementary Tables S8**-**10**).

In this organ modulation of gene expression was higher in the non-vaccinated animals (**Figure 4C1-3**). Regarding antiviral response mediated by type I interferon, a higher number of genes were up-regulated in this group (*isg15* with FC values of 0.94 and 0.97 at 24 and 72 hours pi, respectively, and *mx1*, *mx2*, and *mx3* with FC values of 1.38, 1.42, and 1.11 at 24 hours pi) compared to the vaccinated fish (*isg15* and *mx1* with FC values of 0.78 and 1.87 at 24 hours pi). Interestingly, in both groups, a strong down-regulation of *ifi30* was detected throughout the experiment. Contrary to this, in mock-vaccinated animals, no up-regulation of genes related to this pathway was observed. Moreover, a down-regulation of *pkr*, *mx2*, and *mx3* was registered at 48 hours pi pointing out an inhibited antiviral response in this fish group (FC values of -0.55, -1.59, and -0.98, respectively) (**Supplementary Tables S8**-**10**).

Regarding inflammatory process, major down-regulation of genes was detected (*il8*, *ck3*, *ck8*, and *ck10* in the non-vaccinated; *il8*, *ck3*, *ccr3*, *ck10*, and *cox2* in the vaccinated fish; *il8*, *tnfα*, *ck3*, *ccr3*, *ck10*, and *elam* in the mock-vaccinated group) (**Table 3**), although at 72 hours pi in non-vaccinated animals up-regulation of *il1β*, *il10*, *csf1r*, and *ncf4* (FC values of 2.41, 1.48, 0.96, and 1.04, respectively) was registered. Interestingly, in vaccinated fish, the detection of *csf1r* (FC of 0.58), related to macrophages and inflammatory process, was also detected but early on in the infection (24 hours pi). In addition, *il1β* was also up-regulated in vaccinated fish at the same timepoint as the non-vaccinated fish (FC of 1.06) (**Supplementary Tables S8**-**10**). No gene related to inflammation was up-regulated in the mock-vaccinated fish in this organ.

In non-vaccinated animals, an exclusive up-regulation of *tlr5* (FC of 2.17) was detected at 72 hours pi, as described previously in the spleen samples. At the same timepoint genes coding for c-type lectin 10a (*clec10a*), related to modulation of the immune response, the humoral marker *ilc*, and *hsp70*, related to molecular stress response, were also up-regulated (FC values of 2.3, 1.28, and 0.69, respectively). Regarding non-specific cellular-mediated response, early on in the infection, the up-regulation of *nccrp1* (FC of 0.59) was observed. However, the expression of the *nccrp1* was inhibited later on (FC of -0.84) at 48 hours pi. This profile of expression changes in the vaccinated fish where the up-regulation remained from 24 to 48 hours pi (FC values of 0.58 and 0.82, respectively). In contrast, no modulation of this gene was observed in the mock group. However, an exclusive up-regulation of *perp*, related to cellular apoptosis, was detected at 24 hours pi (FC of 0.7) in mock-vaccinated fish (**Supplementary Tables S8**-**10**).

#### 3.2.4 DEGs in caudal fin

In caudal fin samples, 32 DEGs were identified through the experiment. The clustering analysis of the different samples showed the same profile that was obtained in the spleen, with samples of all experimental groups establishing a cluster at 24 hours pi, and the differentiation of samples for the vaccinated and mock-vaccinated fish at 72 hours pi (**Figure 3D**). Chemokine 10 (*ck10*) gene registered the lowest fold change value in this organ in non-vaccinated animals (-1.88 at 72 hours pi). In contrast, the MX dynamin Like GTPase 3 (*mx3*) gene had the highest fold change value in vaccinated fish (2.28 at 72 hours pi) (**Supplementary Tables S11**-**13**).

In non-vaccinated animals, the up-regulation of genes related to type I IFN (*pkr*, with FC of 0.5), inflammation (*il1β*, with FC of 0.95), B-cell markers (*cd48*, with FC of 0.54), non-specific cell-mediated response (*nccrp1*, with FC of 1.09), and cellular apoptosis (*casp1* and *perp*, with FC values of 0.56 and 0.7, respectively) were detected at 24 hours pi. In ulterior times post-infection, all DEGs were down-regulated in this fish group (**Figure 4D1-3**, **Table 3**, **Supplementary Table S11**). In contrast, in vaccinated fish a clearly different profile of expression was observed, with major down-regulation at 24 hours pi. Regarding type I IFN response, the exclusive up-regulation of *isg15*, *mx1*, *mx2*, and *mx3* (FC values of 1.27, 1.06, 1.03, and 2.28, respectively) at 72 hours pi was remarkable. In addition, cellular apoptosis was mediated by *casp1* up-regulation, which occurred in non-vaccinated fish, but at a later point in time (48 hours pi) (**Figure 4D1-3**, **Table 3**, **Supplementary Table S12**). The immune response studied in the mock group revealed a different profile of expression compared to the other groups and was characterized by the induction of *isg15* (FC of 0.81), and the pro-inflammatory interleukin 8 (*il8*, FC of 0.97) at 48 hours pi. Chemokine 3 receptor (*ccr3*) and chemokine 10 (*ck10*) were also up-regulated (FC values of 0.6 and 1.02, respectively) at 24 hours pi; however at 48 hours pi their tendency of expression changed to down-regulation (FC values of -1.58 and-1.41, respectively). At the same timepoint, *hsp70*, which is related to the stress response, was also induced (FC of 0.53) in this group but only where the expression of this gene was modulated during the experiment in caudal fin samples (**Figure 4D1-3**, **Table 3**, **Supplementary Table S13**).

## 4 Discussion

The study demonstrated that the DNA vaccine (pcDNA-MCP) when administrated to gilthead seabream juveniles one-month before experimental challenge, was able to significantly reduce (*p*<0.05) the viral load and expression of LCDV-Sa in the target site of viral replication (caudal fin) in vaccinated fish compared to that of fish who were not vaccinated or inoculated with an empty plasmid (pcDNA). Both, vaccine and viral inoculation were performed by intramuscular injection. The efficacy of this route of administration was previously reported for vaccine and viral dissemination in fish species (17, 20, 30). Other studies have verified the remarkable efficacy of protection against viral diseases for DNA vaccines administered intramuscularly (31–33).

The immune response of gilthead seabream against LCDV-Sa infection and after a vaccination trial with the pcDNA-MCP has been previously described in head-kidney and intestine samples (17, 20). The profile of the gene expression was different in the infection and vaccination trial groups. Inhibition of the inflammatory process, antigen processing and presentation, humoral and cellular response and a slight activation of the type I interferon route characterized the host response against the viral infection, which was proposed as the cause of LCDV-Sa chronic infection in aquaculture facilities (17). By contrast, for the vaccination trial, an up-regulation of genes related to inflammation was postulated as being responsible for the reduction of viral replication, acting as a marker of vaccine protection efficacy (20). In the present study, we evaluated the immune response in infected-gilthead seabream juveniles that were vaccinated one month prior to the challenge. Head-kidney, spleen, intestine, and caudal fin samples were analyzed, covering a wider spectrum of immune organs and the target tissue for the virus.

Viral infections trigger local and systemic inflammatory responses in the host, recruiting immune cells for adaptive (lymphocytes) and innate immunity (neutrophils, monocytes, and NK cells). Virus recognition by cellular sensors initiates the transcription of pro-inflammatory cytokines, including type I IFN, inducing the synthesis of ISGs as antiviral effector proteins and regulators of immunity (34). In addition, the transcription of toll-like receptors *tlr5* and *tlr9*, and the c-type lectin *cd209* genes were analyzed to evaluate its implication in LCDV-Sa, vaccine, or plasmid recognition by immune cells. It is well known that both, TLR5 and TLR9, have similar functions to the mammalian TLRs (35). Interestingly, different profiles of expression were observed for the different nucleic acid sensors analyzed in the different samples. Regarding TLR9, it has been described as having a higher presence in the spleen compared to other fish organs of different fish, such as zebrafish (*Danio rerio*), Atlantic salmon (*Salmo salar*), rainbow trout (*Oncorhynchus mykiss*), and cobia (*Rachycentron canadum*) (36–39). Up-regulation of *tlr9* only occurred in the spleen of vaccinated (24 hours pi) and mock-vaccinated (72 hours pi) gilthead seabream, while up-regulation of *tlr5* was observed in head-kidney, spleen, and intestine samples of non-vaccinated fish and the head-kidney of mock animals. Similar results were obtained in a previous study using the same model of pathogen-host interaction, where *tlr9* was not differentially expressed in the head-kidney and, on the contrary, *tlr5* was upregulated in head-kidney and intestine of LCDV-Sa-infected gilthead seabreams (17). However, in vaccinated fish no modulation (up or down-regulation) of *tlr5* was detected in any sample analyzed, indicating specific immune induction of toll-like receptors for the different experimental groups. TLR9 has been previously associated with the recognition of different dsDNA viruses (40), including the human cytomegalovirus whose promoter is present in the vaccine vector (pcDNA). Regarding TLR5, it was primarily associated with the detection of bacterial flagellin, however different studies have described the role of this receptor in the reactivation of persisting ranavirus infection through bacterial coinfections (41). Therefore, overexpression of the *mcp* gene in vaccinated fish appears to cause differential expression of pathogen recognition-related genes and could indicate an mcp-independent mechanism of LCDV-Sa entry and recognition through TLR5 regulation. A down-regulation of the expression of CD209, a c-type lectin receptor found specifically in dendritic cells (DC), was observed only in the head-kidney of vaccinated animals early on infection in addition to the *tlr9* inhibition. It has been described that viral recognition by c-type lectin receptors (CTLRs) could favor infection by different viruses (42), therefore its inhibition in vaccinated fish could have a protective role for gilthead seabreams during LCDV-Sa infection.

Regarding inflammation related-gene regulation and IFN response, the specific profiles of gene expression were obtained for the non-vaccinated, vaccinated, and mock fish in an organ-dependant manner. In the head-kidney samples of non-vaccinated animals, inhibition of the inflammatory process and slight IFN response was recorded with the down-regulation of *ck3* (24 and 72 hours pi) and *ck8* (24 hours pi), and only the up-regulation of *irf9*, *mx2* (24 h pi), and *isg15* (72 hours pi). In contrast, in vaccinated fish from 48 hours pi a high pro-inflammatory response mediated by the up-regulation of *il1β* and *il6* transcripts was detected. At the same timepoint, the up-regulation of the c-type lectin domain family 10 member A (*clec10a*) transcript was detected. This receptor was related to the regulation of adaptive and innate immune responses and the induction of the synthesis of several pro-inflammatory cytokines in rainbow trout macrophages and fibroblast-like cells (43). Moreover, at 72 hours pi a higher IFN response was registered through the up-regulation of *irf3*, *isg15*, *mx1*, *mx2*, and *mx3* genes. Nevertheless, the induction of IFN genes was also detected in the mock fish, without the up-regulation of *clec10a* and pro-inflammatory interleukins, establishing a possible role for the vector (pcDNA) as an adjuvant of IFN response induction in this organ. In mammals, it has been described as a “built-in” adjuvant effect derived from CpG-motifs and double-strand DNA for DNA plasmids which are detected by toll-like receptor 9 (TLR9), stimulating IFN-γ-secreting cells in TLR9 +/+ mice but also in TLR9 -/-, suggesting that DNA vaccines induce immune responses by multiple mechanisms different from TLR9 following DNA immunization (44). In the head-kidney samples of mock fish, the *tlr9* transcript was down-regulated. Moreover, the adjuvant properties of the backbone plasmid pcDNA3.3 compared to the plasmid encoding the envelope glycoprotein, hemagglutinin esterase (pHE), of ISAV was able to induce IFN-I response at a higher level in cells (45). In addition, it has been described to have an adjuvant effect of the CpG-enriched plasmid DNA pcDNA3.1 (used in this study) co-administrated with the inactivated grass carp reovirus (GCRV) vaccine in grass carp fingerlings, providing increased levels of IgM in serum, spleen, and head-kidney, as well as up-regulation of *tlr9* and *mx2* expression, inhibiting GCRV proliferation (46). In spleen samples, inflammation and IFN response took place in all experimental groups. However, the timepoint of IFN response diverged between them and different effectors were recorded regarding inflammation. In non-vaccinated animals, high IFN-related transcripts were up-regulated at 24 hours pi, and only *il1β* was up-regulated at 72 hours pi. In vaccinated fish, although the IFN response was less intense, it was prolonged in time. In turn, the only gene associated with inflammation that was overregulated was *elam*. The cytokine TNF-α and il1β released by resident cells are known to induce E-selectin and other chemokines in teleost (47). Endothelial cell leukocyte adhesion molecule-1 (ELAM-1) or E-selectin has been described as an inducible endothelial cell-adhesion molecule for neutrophils and memory T-cells, related to extravasation of these cells at sites of acute inflammation (48). In mammals, leucocytes are maturated in the secondary lymphoid organs, waiting to be recruited by the immune system. In gilthead seabream, the head-kidney has been described as a major hematopoietic and lymphoid organ, with a role in the migration of leucocytes to injured locations (49). Therefore, the up-regulation of *elam* in the present study could indicate a role for the spleen, the secondary lymphoid organ, in the recruitment of leucocytes in this fish species. On the other hand, in mock fish, the IFN response was only observed at 72 hours pi and the unique inflammatory effector detected was interleukin 8 (*il8*). The DEGs detected in the intestinal samples showed the opposite expression trend to that described for the head-kidney, as part of which the interferon response was more intense and took place earlier in the non-vaccinated fish compared to other groups that received the vaccine or the empty plasmid. In addition, the profile of upregulated genes related to inflammation was broader (*il1β*, *il10*, *csf1r*, and *ncf4*) compared to fish inoculated with the DNA plasmid (*csf1r* and *il1β*). This establishes the differential function of the immune organs. However, it is interesting to note that in vaccinated fish the inflammation-related gene *csf1r* was up-regulated early (24 hours pi) compared to non-vaccinated fish (72 hours pi). Furthermore, in the latter, up-regulation of the anti-inflammatory interleukin il10 was detected at 72 hours pi. The kinase receptor CSF-1R is the receptor found in macrophages for the colony-stimulating factor-1 (CSF-1). These have an important role in homeostasis, providing early defense against pathogens, regulation of immune responses and tissue repair (50). Related to inflammation in teleost, it has been described as playing a direct role in promoting the expression of several cytokines including IL-1, Il-6, IL-8, Il-18, TNFα, and IFN (51, 52). Even though the early expression of this genetic marker for macrophage proliferation was detected in vaccinated fish, no significant differences were detected in comparison with the non-vaccinated fish related to cytokine production in the intestine samples. The possible implication of this early expression of *csf1r* in the intestine and the exclusive pro-inflammatory response detected in the head-kidney of vaccinated fish 24 hours later remains uncertain. However, it has been identified as a soluble form of CSF-1R in goldfish (*Carassius auratus* L.), which regulates the CSF-1 activity (53), and promotes the proliferation of kidney primary macrophages (54). In addition, this soluble factor was found in the serum of goldfish giving it a role in systemic regulation of this activity (53). Finally, in caudal fin samples, it is worth noting the exclusive induction in vaccinated fish of the interferon-mediated antiviral response at 72 hours pi (*isg15*, *mx1*, *mx2*, and *mx3*), which was very scarce in non-vaccinated fish and those inoculated with the empty plasmid (*pkr* at 24 hours pi and *isg15* at 48 hours pi, respectively). The differential expression of the pro-inflammatory caspase-1 (*casp1*) and the no-modulation of the expression of the different chemokines analyzed could be the crucial factor that differentiates the results obtained from those of the non-vaccinated group, where a strong anti-inflammatory response by down-regulation of *ccr3*, *ck3*, and *ck10* was detected at 48 and 72 hours pi. This down-regulation of chemokines could inhibit the early inflammatory response mediated by the up-regulation of *casp1* and *perp* detected in the same group at 24 hours pi. In the mock fish, this kind of inflammation-related response was observed by the early up-regulation of *ccr3* and *ck10* and subsequent up-regulation of *isg15* and *il8* 24 h later (48 hours pi). At 48 hours pi, chemokines were down-regulated and no inflammatory or interferon responses were observed later on. The antiviral role of the inflammatory response mediated by chemokines in gilthead seabream has been studied under nodavirus infection in the target site (brain) of viral replication, and a strong up-regulation of CK3, CK8, and CK10, among others chemokines, was correlated with antiviral defense in seabream (55). It seems that the infection by LCDV-Sa in gilthead seabream triggers a different profile of chemokine expression that could be related to the immune evasion mechanisms of the virus at the target site of replication. However, the administration of the vaccine seems to compensate for the lack of chemokine-mediated inflammation through the caspase-1 pathway, triggering an efficient antiviral interferon response.

In terms of antigen presentation and humoral and cellular responses, a major trend in gene dysregulation was detected in the different experimental groups. Regarding hematopoietic organs, in vaccinated gilthead seabream, early expression of recombination activating gene 1 (*rag1*) (24 hours pi), compared to non-vaccinated fish (72 hours pi), and a cluster of differentiation 48 (*cd48*) genes (48 hours pi) were detected in head-kidneys. The *rag1* gene encodes a protein with endonuclease activity related to the assembly of the diversity of immunoglobulins and T cell receptor genes (56) and serves as a marker for the development of the adaptive immune response. It has been postulated that the expression of *rag* genes is crucial to the maturation of B cells and the production of Ig, and its expression within a lymphoid organ can be used as a developmental marker to assess immunological competence (57). Moreover, the cell-surface receptor CD48 is a lipid-anchored protein expressed on all antigen-presenting cells and T cells that contributes to maintaining the inflammatory response (58). As described earlier, at 48 hours pi the up-regulation of *il1β* and *il6* was observed exclusively in the head-kidney of vaccinated fish. Therefore, based on the early expression of these genes in the vaccinated animals, an immunocompetent state provoked by the DNA vaccine could be postulated. In addition, in spleen samples, the markers of humoral response *ighm* (also detected in the other experimental groups) and the immunoglobulin light chain (*ilc*) gene were differentially expressed at 48 hours pi. However, no up-regulation of genes was observed in the intestine and caudal fin, defining an organ-specific immune response in gilthead seabream. Interestingly, in these latter organs, in the non-vaccinated group, the *ilc* (intestine) and *cd48* (caudal fin) genes were detected. The expression of the T-cell receptor (*tcrα*) was only detected in the mock fish. This impaired T-specific cell immunity in contrast to the promotion of humoral response and has been previously described in gilthead seabream infected with LCDV-Sa (17).

Regarding the elimination of infected cells by cell-mediated response, two main genes were detected, the non-specific cytotoxic cell receptor (*nccrp1*) and perforin (*prf1*), which are previously described as crucial effectors for virus clearance. Major differences were observed regarding the non-specific cytotoxic response mediated by the *nccrp1* gene in the different experimental groups. In non-vaccinated fish no modulation of the gene was recorded in hematopoietic organ samples and an up-regulation was detected in vaccinated and mock fish; however, it was only present in vaccinated fish early on in infection. In contrast, in intestine samples, this gene was up-regulated in non-vaccinated and vaccinated fish exclusively, but in the latter, its up-regulation was maintained for a longer period. However, in caudal fin samples, *nccrp1* gene was exclusively up-regulated in non-vaccinated fish. The importance of the innate immune response of this cytotoxic cell effector has been described in gilthead seabream against LCDV (16) and nodavirus (59) infection, as it mediates the leucocyte killing of virus-infected cells. Genes related to apoptosis were scarcely modulated through the experiment. Only in spleen samples, *prf1* gene up-regulation was remarkable, especially in vaccinated fish at 72 hours pi, which was the transcript with the highest fold change values registered in the experiment, although it was also detected in mock fish at the same timepoint but with lower expression. Perforin gene has been identified in several teleost fish, including zebrafish (60), Japanese flounder (*Paralichthys olivaceus*) (61), rainbow trout (62), rock bream (*Oplegnathus fasciatus*) (63), ginbuna crucian carp (*Carassius auratus langsdorfii*) (64), and common carp (*Cyprinus carpio*) (65). Similar to mammals, perforin is involved in the immune defense against virus infections in teleosts. Perforin, a pore-forming glycoprotein, has been demonstrated to play key roles in clearing virus-infected cells, also playing indispensable roles in CD8+T cell-mediated cytotoxicity (64). This is the first study to analyse the involvement of perforin in the immune response of gilthead seabream after infection with LCDV. Interestingly, in the head-kidney samples, the main immune organ in teleost fish (66), no differential expression of *prf1* was detected. The up-regulation of perforin genes has been described after infection with different viruses (60, 63, 65, 67, 68) and has mainly been studied in kidney samples where the modulation occurred several days after infection, which could explain the results of this study, establishing specific immune roles in a time-dependant manner against pathogens between the two hematopoietic organs analyzed.

In conclusion, our data suggest that the administration of the DNA vaccine (pcDNA-MCP) in gilthead seabream juveniles reduces the viral replication after inoculation of fish with LCDV-Sa. In addition, specific immune determinants have been detected exclusively in vaccinated fish that could be related to this control of viral multiplication. The specific role of the immune response of each of the organs analyzed has been denoted. An early humoral and cellular response mediated by *rag1* and *cd48*, and a pro-inflammatory response mediated by *il1β* and *il6* in head-kidney were also observed. This could be related to the possible presence of a soluble form of macrophage receptor (CSF-1R) found in intestine samples. In addition to a specific modulation of toll-like receptor 9 (*tlr9*), a recruitment of leukocytes by overexpression of *elam* and a cell-mediated cytotoxic response controlled by *nccrp1* and *prf1* was detected in the spleen. Moreover, an efficient antiviral response was detected through the interferon effectors *isg15*, *mx1*, *mx2*, and *mx3* in the target site of viral replication in the context of lymphocystis disease. More comparative studies examining the route of administration by oral chitosan beads containing the vaccine will be carried out. This study furthers understanding of the immune determinants modulated in vaccinated gilthead seabream following infection with LCDV-Sa, outlining which could confer protection against this viral disease for the aquaculture sector.

## Data availability statement

The original contributions presented in the study are included in the article/**Supplementary Material**, further inquiries can be directed to the corresponding author/s.

## Ethics statement

The animal study was reviewed and approved by Spanish authorities for the regulation of animal care and experimentation.

## Author contributions

AL, RL-R, JB, and DC conceived and designed the study. RL-R and AL performed the experimental trials. AL, RL-R, and JG-M carried out all gene expression experiments and data analyses. AL and RL-R wrote the manuscript. JB and DC revised the manuscript. All authors contributed to the article and approved the submitted version.
