# Supplementary material for: Immune response of DNA vaccinated-gilthead seabream (Sparus aurata) against LCDV-Sa infection: relevance of the inflammatory process
Source: Front Immunol. 2023 Jun 6;14:1209926. doi: 10.3389/fimmu.2023.1209926 (PMC10279854; doi:10.3389/fimmu.2023.1209926)
Supplement: Supplementary file 2 [file Table_2.docx]

**Supplementary Table S2**. Differentially expressed genes (DEGs) in head-kidney from non-vaccinated animals.

| Biological process | Gene | Head-kidney | | | | | | | | |
| --- | --- | --- | --- | --- | --- | --- | --- | --- | --- | --- |
|  |  |  |  |  |  |  |  |  |  |  |
|  |  | 24 h pi | | | 48 h pi | | | 72 h pi | | |
|  |  | FC | Log_2_FC | *p*-value | FC | Log_2_FC | *p*-value | FC | Log_2_FC | *p*-value |
| Viral recognition | | | | | | | | | | |
|  | *tlr9* | 0.33 | **-1.60** | 0.0006 | 0.65 | -0.61 | 0.14 | 0.76 | -0.38 | 0.3 |
|  | *tlr5* | 2.07 | **1.05** | 0.00095 | 0.87 | -0.19 | 0.5 | 1.4 | 0.49 | 0.13 |
|  | *cd209* | 0.85 | -0.23 | 0.4 | 0.73 | -0.44 | 0.19 | 1.06 | 0.09 | 0.7 |
| Regulation of innate and adaptive immune response | |  | | | | | | | | |
|  | *clec10a* | 0.81 | -0.3 | 0.6 | 0.93 | -0.1 | 0.79 | 1.19 | 0.25 | 0.5 |
|  | *tgfβ1* | 0.61 | **-0.71** | 0.011 | 0.95 | -0.06 | 0.8 | 1.0 | 0.005 | 0.9 |
| Type I IFN response | |  | | | | | | | | |
|  | *irf1* | 0.67 | **-0.58** | 0.037 | 0.87 | -0.19 | 0.5 | 0.9 | -0.14 | 0.6 |
|  | *irf3* | 1.58 | 0.66 | 0.2 | 0.7 | -0.3 | 0.2 | 0.96 | -0.056 | 0.7 |
|  | *irf9* | 7.56 | **2.92** | 0.031 | 0.3 | -1.7 | 0.1 | 1.6 | 0.68 | 0.6 |
|  | *isg15* | 2.03 | 1.02 | 0.2 | 0.8 | -0.3 | 0.6 | 1.91 | **0.93** | 0.04 |
|  | *mx1* | 0.76 | -0.38 | 0.4 | 0.6 | -0.63 | 0.1 | 1.5 | 0.6 | 0.6 |
|  | *mx2* | 1.5 | **0.58** | 0.036 | 0.7 | -0.4 | 0.3 | 1.74 | 0.8 | 0.1 |
|  | *mx3* | 1.41 | 0.49 | 0.12 | 1.19 | 0.25 | 0.6 | 1.3 | 0.41 | 0.3 |
|  | *ifi30* | 0.5 | -0.9 | 0.08 | 0.93 | -0.1 | 0.7 | 1.1 | 0.15 | 0.5 |
| Inflammatory process | |  | | | | | | | | |
|  | *il1β* | 0.52 | 0.93 | 0.3 | 1.19 | 0.16 | 0.8 | 1.49 | 0.58 | 0.4 |
|  | *il6* | 0.9 | -0.14 | 0.59 | 0.7 | -0.3 | 0.45 | 0.83 | -0.26 | 0.6 |
|  | *il8* | 0.82 | -0.27 | 0.6 | 1.26 | 0.33 | 0.3 | 1.26 | 0.33 | 0.5 |
|  | *ck3* | 0.33 | **-1.60** | 0.008 | 0.5 | **-0.985** | 0.038 | 0.72 | -0.45 | 0.3 |
|  | *ccr3* | 0.39 | -1.3 | 0.15 | 0.85 | -0.23 | 0.4 | 1.07 | 0.1 | 0.6 |
|  | *ck8* | 0.44 | **-1.18** | 0.002 | 0.85 | -0.22 | 0.4 | 1.3 | 0.4 | 0.18 |
|  | *csf1r* | 0.55 | -0.86 | 0.12 | 0.9 | -0.14 | 0.7 | 1.0 | 0.001 | 0.9 |
| Antigen processing and presentation | |  | | | | | | | | |
|  | *iclp* | 0.55 | **-0.86** | 0.044 | 0.76 | -0.39 | 0.35 | 0.93 | -0.095 | 0.74 |
|  | *mrc1* | 0.33 | -1.5 | 0.17 | 1.01 | 0.027 | 0.92 | 0.97 | -0.043 | 0.87 |
| Humoral and cellular immune response | |  | | | | | | | | |
|  | *tcrα* | 0.75 | -0.39 | 0.2 | 0.79 | -0.32 | 0.39 | 1.2 | 0.28 | 0.14 |
|  | *tcrβ* | 0.66 | -0.58 | 0.2 | 0.40 | **-1.32** | 0.04 | 1.3 | 0.38 | 0.22 |
|  | *rag1* | 1.7 | 0.76 | 0.15 | 1.52 | 0.6 | 0.085 | 2.32 | **1.21** | 0.002 |
|  | *cd48* | 0.68 | -0.54 | 0.6 | 0.96 | -0.05 | 0.92 | 0.85 | -0.23 | 0.48 |
|  | *cd276* | 0.98 | -0.025 | 0.9 | 0.62 | **-0.68** | 0.035 | 1.22 | 0.29 | 0.3 |
| Nonspecific cytotoxic cell receptor | |  |  |  |  |  |  |  |  |  |
|  | *nccrp1* | 1.5 | 0.59 | 0.7 | 1.28 | 0.36 | 0.44 | 1.11 | 0.15 | 0.59 |
| Proteolysis and apoptotic process | |  | | | | | | | | |
|  | *ctsb* | 0.63 | -0.66 | 1.6 | 0.94 | -0.085 | 0.85 | 1.01 | 0.16 | 0.95 |
|  | *casp1* | 0.66 | **-0.60** | 0.015 | 0.81 | -0.28 | 0.3 | 1.05 | 0.072 | 0.73 |
|  | *lgals1* | 0.21 | **-2.25** | 0.02 | 0.83 | -0.26 | 0.49 | 1.09 | 0.12 | 0.66 |
|  | *perp* | 0.89 | -0.16 | 0.6 | 0.82 | -0.28 | 0.31 | 1.52 | **0.6** | 0.012 |
| Molecular stress response | |  | | | | | | | | |
|  | *hsp90* | 0.7 | **-0.51** | 0.043 | 0.89 | -0.15 | 0.39 | 1.1 | 0.22 | 0.2 |

**Supplementary Table S3.** Differentially expressed genes (DEGs) in head-kidney from vaccinated animals.

| Biological process | Gene | Head-kidney | | | | | | | | |
| --- | --- | --- | --- | --- | --- | --- | --- | --- | --- | --- |
|  |  |  |  |  |  |  |  |  |  |  |
|  |  | 24 h pi | | | 48 h pi | | | 72 h pi | | |
|  |  | FC | Log_2_FC | *p*-value | FC | Log_2_FC | *p*-value | FC | Log_2_FC | *p*-value |
| Viral recognition | | | | | | | | | | |
|  | *tlr9* | 0.4 | **-1.32** | 0.0003 | 0.78 | -0.34 | 0.32 | 0.68 | -0.54 | 0.2 |
|  | *tlr5* | 1.22 | 0.29 | 0.2 | 1.2 | 0.26 | 0.38 | 1.04 | 0.067 | 0.8 |
|  | *cd209* | 0.51 | **-0.97** | 0.007 | 0.94 | -0.082 | 0.75 | 0.62 | -0.67 | 0.17 |
| Regulation of innate and adaptive immune response | |  | | | | | | | | |
|  | *clec10a* | 1.44 | 0.52 | 0.4 | 2.78 | **1.48** | 0.044 | 0.87 | -0.19 | 0.72 |
|  | *tgfβ1* | 0.6 | **-0.74** | 0.005 | 0.8 | -0.3 | 0.26 | 0.71 | -0.8 | 0.56 |
| Type I IFN response | |  | | | | | | | | |
|  | *irf1* | 0.6 | **-0.74** | 0.006 | 0.83 | -0.26 | 0.5 | 0.72 | -0.47 | 0.15 |
|  | *irf3* | 1.28 | 0.36 | 0.2 | 0.78 | -0.34 | 0.1 | 1.65 | **0.72** | 0.012 |
|  | *irf9* | 1.15 | 0.20 | 0.6 | 0.41 | -1.2 | 0.2 | 0.76 | -0.58 | 0.68 |
|  | *isg15* | 1.5 | 0.58 | 0.3 | 0.48 | -1.05 | 0.56 | 1.81 | **0.86** | 0.002 |
|  | *mx1* | 1.2 | 0.26 | 0.5 | 0.9 | -0.14 | 0.62 | 3.79 | **1.92** | 0.0001 |
|  | *mx2* | 1.53 | 0.62 | 0.2 | 0.87 | -0.19 | 0.25 | 3.7 | **1.89** | 0.001 |
|  | *mx3* | 1.07 | 0.09 | 0.7 | 1.38 | 0.46 | 0.3 | 4.02 | **2.01** | 0.008 |
|  | *ifi30* | 0.53 | **-0.92** | 0.0002 | 1.23 | 0.3 | 0.28 | 0.96 | -0.054 | 0.83 |
| Inflammatory process | |  | | | | | | | | |
|  | *il1β* | 0.23 | -2.0 | 0.8 | 4.14 | **2.05** | 0.03 | 1.24 | 0.31 | 0.52 |
|  | *il6* | 0.99 | 0.015 | 0.9 | 1.81 | **0.86** | 0.048 | 2.17 | 1.12 | 0.93 |
|  | *il8* | 0.51 | -0.97 | 0.08 | 1.15 | 0.2 | 0.69 | 1.03 | 0.052 | 0.9 |
|  | *ck3* | 0.23 | **-2.12** | 0.0005 | 0.67 | -0.57 | 0.95 | 0.75 | -0.4 | 0.5 |
|  | *ccr3* | 0.45 | **-1.15** | 0.0001 | 0.93 | -0.09 | 0.69 | 0.65 | -0.61 | 0.06 |
|  | *ck8* | 0.52 | **-0.94** | 0.018 | 1.03 | 0.05 | 0.87 | 0.87 | -0.19 | 0.7 |
|  | *csf1r* | 0.66 | **-0.6** | 0.031 | 1.2 | 0.28 | 0.4 | 0.82 | -0.28 | 0.35 |
| Antigen processing and presentation | |  | | | | | | | | |
|  | *iclp* | 0.75 | -0.4 | 0.07 | 1.04 | 0.063 | 0.81 | 0.98 | -0.019 | 0.95 |
|  | *mrc1* | 0.44 | **-1.18** | 0.0004 | 1.094 | 0.13 | 0.54 | 0.69 | -0.53 | 0.66 |
| Humoral and cellular immune response | |  | | | | | | | | |
|  | *tcrα* | 0.69 | **-0.54** | 0.017 | 0.81 | -0.30 | 0.37 | 1.12 | 0.16 | 0.78 |
|  | *tcrβ* | 0.78 | -0.35 | 0.4 | 0.94 | -0.08 | 0.77 | 1.4 | 0.54 | 0.46 |
|  | *rag1* | 1.9 | **0.93** | 0.016 | 1.46 | 0.54 | 0.13 | 3.5 | 1.82 | 0.097 |
|  | *cd48* | 0.55 | **-0.86** | 0.025 | 1.637 | **0.71** | 0.011 | 0.74 | -0.42 | 0.18 |
|  | *cd276* | 0.75 | -0.41 | 0.1 | 0.82 | -0.27 | 0.44 | 0.95 | -0.06 | 0.83 |
| Nonspecific cytotoxic cell receptor | |  | | | | | | | | |
|  | *nccrp1* | 2.2 | 1.1 | 0.4 | 2.176 | **1.12** | 0.011 | 1.07 | 0.098 | 0.75 |
| Proteolysis and apoptotic process | |  | | | | | | | | |
|  | *ctsb* | 0.58 | **-0.79** | 0.024 | 0.9 | -0.14 | 0.75 | 0.87 | -0.19 | 0.54 |
|  | *casp1* | 0.74 | -0.41 | 0.6 | 1.19 | 0.25 | 0.19 | 0.97 | -0.03 | 0.89 |
|  | *lgals1* | 0.27 | **-1.89** | 0.00007 | 1.05 | 0.083 | 0.81 | 0.63 | -0.76 | 0.83 |
|  | *perp* | 0.79 | -0.33 | 0.2 | 0.92 | -0.11 | 0.62 | 1.23 | 0.3 | 0.12 |
| Molecular stress response | |  | | | | | | | | |
|  | *hsp90* | 0.7 | **-0.51** | 0.021 | 0.97 | -0.03 | 0.83 | 0.82 | -0.27 | 0.13 |

**Supplementary Table S4.** Differentially expressed genes (DEGs) in head-kidney from mock-vaccinated animals.

| Biological process | Gene | Head-kidney | | | | | | | | |
| --- | --- | --- | --- | --- | --- | --- | --- | --- | --- | --- |
|  |  |  |  |  |  |  |  |  |  |  |
|  |  | 24 h pi | | | 48 h pi | | | 72 h pi | | |
|  |  | FC | Log_2_FC | *p*-value | FC | Log_2_FC | *p*-value | FC | Log_2_FC | *p*-value |
| Viral recognition | | | | | | | | | | |
|  | *tlr9* | 0.69 | -0.5 | 0.14 | 0.58 | **-0.775** | 0.018 | 1.29 | 0.37 | 0.36 |
|  | *tlr5* | 1.58 | **0.66** | 0.011 | 1.46 | 0.55 | 0.14 | 0.98 | -0.028 | 0.93 |
|  | *cd209* | 0.74 | -0.42 | 0.9 | 0.75 | -0.4 | 0.3 | 1.0 | 0.035 | 0.9 |
| Regulation of innate and adaptive immune response | |  | | | | | | | | |
|  | *clec10a* | 0.87 | -0.18 | 0.8 | 0.85 | -0.22 | 0.68 | 1.31 | 0.39 | 0.55 |
|  | *tgfβ1* | 0.67 | **-0.58** | 0.015 | 1.05 | 0.075 | 0.76 | 1.1 | 0.14 | 0.62 |
| Type I IFN response | |  | | | | | | | | |
|  | *irf1* | 0.55 | **-0.86** | 0.005 | 0.96 | -0.04 | 0.86 | 0.86 | -0.2 | 0.55 |
|  | *irf3* | 0.81 | -0.29 | 0.2 | 0.94 | -0.07 | 0.73 | 1.49 | **0.57** | 0.042 |
|  | *irf9* | 1.88 | **0.91** | 0.025 | 0.16 | **-2.62** | 0.03 | 1.2 | 0.26 | 0.79 |
|  | *isg15* | 0.82 | -0.27 | 0.4 | 0.78 | -0.34 | 0.51 | 1.74 | **0.8** | 0.015 |
|  | *mx1* | 0.81 | -0.28 | 0.3 | 1.15 | 0.21 | 0.35 | 3.68 | **1.88** | 0.015 |
|  | *mx2* | 0.94 | -0.07 | 0.8 | 1.05 | 0.071 | 0.75 | 4.53 | **2.17** | 0.012 |
|  | *mx3* | 0.79 | -0.3 | 0.2 | 1.65 | 0.72 | 0.06 | 4.48 | **2.16** | 0.005 |
|  | *ifi30* | 0.72 | -0.45 | 0.4 | 1.16 | 0.21 | 0.51 | 2.1 | **1.07** | 0.004 |
| Inflammatory process | |  | | | | | | | | |
|  | *il1β* | 0.16 | -2.6 | 0.08 | 2.4 | 1.2 | 0.21 | 0.66 | -0.5 | 0.26 |
|  | *il6* | 1.09 | 0.13 | 0.7 | 1.0 | 0.07 | 0.08 | 1.38 | 0.46 | 0.66 |
|  | *il8* | 0.42 | **-1.25** | 0.046 | 0.96 | -0.05 | 0.92 | 0.44 | **-1.18** | 0.001 |
|  | *ck3* | 0.41 | **-1.29** | 0.01 | 1.06 | 0.084 | 0.72 | 1.08 | 0.11 | 0.83 |
|  | *ccr3* | 0.55 | **-0.86** | 0.001 | 0.74 | -0.38 | 0.31 | 1.36 | 0.44 | 0.13 |
|  | *ck8* | 0.56 | **-0.84** | 0.045 | 0.93 | -0.1 | 0.73 | 1.3 | 0.38 | 0.26 |
|  | *csf1r* | 0.89 | -0.16 | 0.4 | 1.23 | 0.3 | 0.39 | 1.08 | 0.11 | 0.59 |
| Antigen processing and presentation | |  | | | | | | | | |
|  | *iclp* | 0.78 | -0.34 | 0.16 | 0.93 | -0.09 | 0.72 | 1.12 | 0.17 | 0.59 |
|  | *mrc1* | 0.55 | **-0.86** | 0.004 | 1.01 | 0.017 | 0.96 | 1.3 | 0.38 | 0.18 |
| Humoral and cellular immune response | |  |  |  |  |  |  |  |  |  |
|  | *tcrα* | 0.87 | -0.19 | 0.4 | 0.99 | -0.09 | 0.97 | 0.69 | -0.05 | 0.12 |
|  | *tcrβ* | 0.83 | -0.26 | 0.5 | 0.93 | -0.09 | 0.77 | 1.14 | 0.18 | 0.54 |
|  | *rag1* | 1.04 | 0.05 | 0.8 | 1.54 | 0.62 | 0.29 | 1.27 | 0.34 | 0.4 |
|  | *cd48* | 0.73 | -0.43 | 0.1 | 1.21 | 0.28 | 0.35 | 1.1 | 0.14 | 0.69 |
|  | *cd276* | 1.0 | 0.003 | 0.9 | 0.9 | -0.15 | 0.66 | 1.1 | 0.22 | 0.45 |
| Nonspecific cytotoxic cell receptor | |  | | | | | | | | |
|  | *nccrp1* | 2.84 | 1.5 | 0.3 | 1.7 | 0.77 | 0.15 | 2.18 | **1.12** | 0.044 |
| Proteolysis and apoptotic process | |  | | | | | | | | |
|  | *ctsb* | 0.74 | -0.42 | 0.2 | 0.93 | -0.091 | 0.81 | 0.83 | -0.26 | 0.26 |
|  | *casp1* | 0.85 | -0.22 | 0.2 | 1.09 | 0.12 | 0.6 | 1.11 | 0.16 | 0.59 |
|  | *lgals1* | 0.38 | **-1.4** | 0.001 | 1.03 | 0.051 | 0.92 | 1.88 | **0.91** | 0.043 |
|  | *perp* | 0.91 | -0.12 | 0.6 | 0.86 | -0.2 | 0.43 | 1.05 | 0.08 | 0.79 |
| Molecular stress response | |  |  |  |  |  |  |  |  |  |
|  | *hsp90* | 0.79 | -0.33 | 0.1 | 1.05 | 0.07 | 0.63 | 0.91 | -0.12 | 0.55 |

**Supplementary Table S5.** Differentially expressed genes (DEGs) in spleen from non-vaccinated animals.

| Biological process | Gene | Spleen | | | | | | | |  |
| --- | --- | --- | --- | --- | --- | --- | --- | --- | --- | --- |
|  |  |  | | | | | | | |  |
|  |  | 24 h pi | | | 48 h pi | | | 72 h pi | |  |
|  |  | FC | Log_2_FC | *p*-value | FC | Log_2_FC | *p*-value | FC | Log_2_FC | *p*-value |
| Viral recognition | | | | | | | | | |  |
|  | *tlr9* | 1.32 | 0.4 | 0.19 | 0.95 | -0.07 | 0.87 | 0.95 | -0.07 | 0.91 |
|  | *tlr5* | 1.35 | 0.4 | 0.3 | 1.33 | 0.41 | 0.3 | 3.85 | **1.94** | 0.034 |
| Type I IFN response | |  | | | | | | | |  |
|  | *irf3* | 1.6 | 0.67 | 0.11 | 0.66 | -0.59 | 0.11 | 1.48 | 0.56 | 0.31 |
|  | *irf9* | 4.42 | **2.15** | 0.026 | 0.34 | -1.57 | 0.25 | 2.92 | 1.55 | 0.38 |
|  | *pkr* | 1.75 | **0.81** | 0.046 | 0.93 | -0.11 | 0.79 | 1.3 | 0.38 | 0.42 |
|  | *isg15* | 1.9 | **0.89** | 0.031 | 0.7 | -0.52 | 0.2 | 2.04 | 1.03 | 0.09 |
|  | *mx1* | 2.5 | **1.29** | 0.011 | 0.75 | -0.41 | 0.41 | 2.86 | 1.51 | 0.06 |
|  | *mx2* | 3.4 | **1.74** | 0.028 | 0.64 | -0.64 | 0.13 | 1.53 | 0.62 | 0.43 |
|  | *mx3* | 1.78 | 0.83 | 0.11 | 0.85 | -0.24 | 0.62 | 2.47 | 1.31 | 0.08 |
| Inflammatory process | |  | | | | | | | |  |
|  | *il1β* | 0.44 | -1.19 | 0.25 | 0.98 | -0.002 | 0.97 | 4.16 | **2.06** | 0.039 |
|  | *il8* | 0.82 | -0.28 | 0.75 | 1.24 | 0.31 | 0.63 | 2.4 | 1.26 | 0.27 |
|  | *ck3* | 0.46 | **-1.11** | 0.038 | 0.81 | -0.3 | 0.56 | 1.27 | 0.35 | 0.63 |
|  | *elam* | 1.02 | 0.025 | 0.97 | 2.08 | 1.06 | 0.11 | 1.88 | 0.91 | 0.24 |
| Humoral and cellular immune response | |  | | | | | | | |  |
|  | *tcrα* | 1.0 | 0.007 | 0.98 | 1.25 | 0.32 | 0.48 | 1.55 | 0.63 | 0.37 |
|  | *ighm* | 1.79 | 0.84 | 0.15 | 2.08 | **1.06** | 0.002 | 1.8 | 0.85 | 0.1 |
|  | *ilc* | 1.7 | 0.76 | 0.2 | 1.46 | 0.54 | 0.22 | 1.58 | 0.66 | 0.35 |
| Nonspecific cytotoxic cell receptor | |  |  |  |  |  |  |  |  |  |
|  | *nccrp1* | 1.46 | 0.55 | 0.15 | 0.86 | -0.22 | 0.57 | 1.77 | 0.82 | 0.12 |
| Proteolysis and apoptotic process | |  |  |  |  |  |  |  |  |  |
|  | *lgals1* | 1.33 | 0.41 | 0.11 | 0.83 | -0.26 | 0.39 | 1.35 | 0.43 | 0.35 |
|  | *prf1* | 0.41 | -1.29 | 0.4 | 3.8 | 1.92 | 0.19 | 15.37 | 3.94 | 0.096 |

**Supplementary Table S6.** Differentially expressed genes (DEGs) in spleen from vaccinated animals.

| Biological process | Gene | Spleen | | | | | | | | |
| --- | --- | --- | --- | --- | --- | --- | --- | --- | --- | --- |
|  |  |  |  |  |  |  |  |  |  |  |
|  |  | 24 h pi | | | 48 h pi | | | 72 h pi | | |
|  |  | FC | Log_2_FC | *p*-value | FC | Log_2_FC | *p*-value | FC | Log_2_FC | *p*-value |
| Viral recognition | |  | | | | | | | | |
|  | *tlr9* | 2.05 | **1.03** | 0.0133 | 1.11 | 0.15 | 0.83 | 1.68 | 0.75 | 0.18 |
|  | *tlr5* | 0.74 | -0.43 | 0.15 | 2.04 | 1.03 | 0.18 | 2.07 | 1.05 | 0.14 |
| Type I IFN response | |  |  |  |  |  |  |  |  |  |
|  | *irf3* | 1.3 | 0.38 | 0.26 | 0.81 | -0.31 | 0.28 | 1.66 | 0.73 | 0.12 |
|  | *irf9* | 1.02 | 0.03 | 0.87 | 0.54 | -0.88 | 0.51 | 1.19 | 0.26 | 0.84 |
|  | *pkr* | 1.3 | 0.37 | 0.032 | 1.1 | 0.13 | 0.57 | 1.09 | 0.12 | 0.74 |
|  | *isg15* | 1.53 | 0.61 | 0.09 | 0.88 | -0.19 | 0.71 | 1.17 | 0.22 | 0.64 |
|  | *mx1* | 2.27 | **1.18** | 0.045 | 0.91 | -0.13 | 0.63 | 6.14 | **2.62** | 0.004 |
|  | *mx2* | 3.53 | **1.82** | 0.036 | 0.79 | -0.33 | 0.23 | 4.22 | **2.08** | 0.003 |
|  | *mx3* | 1.5 | 0.58 | 0.29 | 0.91 | -0.14 | 0.56 | 3.13 | **1.65** | 0.0163 |
| Inflammatory process | |  | | | | | | | | |
|  | *il1β* | 0.46 | -1.11 | 0.32 | 2.23 | 1.16 | 0.15 | 1.31 | 0.39 | 0.63 |
|  | *il8* | 0.57 | -0.8 | 0.36 | 3.11 | 1.63 | 0.052 | 0.48 | -1.07 | 0.21 |
|  | *ck3* | 0.5 | **-1.01** | 0.01 | 0.72 | -0.47 | 0.19 | 0.76 | -0.4 | 0.42 |
|  | *elam* | 0.55 | **-0.85** | 0.025 | 3.27 | **1.71** | 0.015 | 0.85 | -0.23 | 0.7 |
| Humoral and cellular immune response | |  | | | | | | | | |
|  | *tcrα* | 0.58 | -0.56 | 0.051 | 1.5 | 0.59 | 0.19 | 0.65 | -0.62 | 0.3 |
|  | *ighm* | 1.55 | 0.63 | 0.11 | 2.53 | **1.34** | 0.002 | 1.35 | 0.43 | 0.32 |
|  | *ilc* | 1.1 | 0.13 | 0.62 | 2.38 | **1.25** | 0.028 | 0.75 | -0.41 | 0.52 |
| Nonspecific cytotoxic cell receptor | |  | | | | | | | | |
|  | *nccrp1* | 1.62 | **0.7** | 0.037 | 1.16 | 0.21 | 0.52 | 1.79 | **0.87** | 0.0236 |
| Proteolysis and apoptotic process | |  |  |  |  |  |  |  |  |  |
|  | *lgals1* | 1.35 | 0.43 | 0.067 | 1.24 | 0.31 | 0.48 | 1.45 | 0.54 | 0.2 |
|  | *prf1* | 0.45 | -1.15 | 0.57 | 13.5 | 3.76 | 0.11 | 77.9 | **6.28** | 1.03E-6 |

**Supplementary Table S7.** Differentially expressed genes (DEGs) in spleen from mock-vaccinated animals.

| Biological process | Gene | Spleen | | | | | | | | |
| --- | --- | --- | --- | --- | --- | --- | --- | --- | --- | --- |
|  |  |  |  |  |  |  |  |  |  |  |
|  |  | 24 h pi | | | 48 h pi | | | 72 h pi | | |
|  |  | FC | Log_2_FC | *p*-value | FC | Log_2_FC | *p*-value | FC | Log_2_FC | *p*-value |
| Viral recognition | | | | | | | | | | |
|  | *tlr9* | 1.84 | 0.88 | 0.051 | 1.03 | 0.05 | 0.92 | 3.44 | **1.78** | 0.001 |
|  | *tlr5* | 0.94 | -0.08 | 0.78 | 1.98 | 0.99 | 0.09 | 0.95 | -0.08 | 0.91 |
| Type I IFN response | |  | | | | | | | | |
|  | *irf3* | 0.96 | -0.05 | 0.78 | 1.21 | 0.28 | 0.33 | 2.21 | **1.14** | 0.047 |
|  | *irf9* | 0.9 | -0.16 | 0.55 | 0.27 | -1.9 | 0.17 | 1.71 | 0.77 | .055 |
|  | *pkr* | 1.22 | 0.29 | 0.15 | 1.18 | 0.24 | 0.26 | 2.94 | **1.55** | 0.012 |
|  | *isg15* | 0.84 | -0.25 | 0.46 | 0.98 | -0.03 | 0.9 | 2.5 | 1.32 | 0.053 |
|  | *mx1* | 1.29 | 0.36 | 0.36 | 1.14 | 0.18 | 0.58 | 8.43 | **3.07** | 0.001 |
|  | *mx2* | 1.57 | 0.65 | 0.37 | 0.87 | -0.19 | 0.69 | 5.22 | **2.38** | 0.003 |
|  | *mx3* | 0.81 | -0.3 | 0.59 | 1.18 | 0.23 | 0.62 | 3.55 | **1.83** | 0.009 |
| Inflammatory process | |  | | | | | | | | |
|  | *il1β* | 0.44 | -1.2 | 0.19 | 1.21 | 0.27 | 0.65 | 0.88 | -0.18 | 0.76 |
|  | *il8* | 0.47 | -1.07 | 0.2 | 1.9 | **0.93** | 0.0418 | 0.36 | -1.45 | 0.09 |
|  | *ck3* | 0.42 | **-1.24** | 0.0219 | 1.02 | 0.03 | 0.89 | 1.76 | 0.82 | 0.17 |
|  | *elam* | 0.68 | -0.56 | 0.09 | 2.14 | 1.1 | 0.051 | 0.44 | -1.2 | 0.1 |
| Humoral and cellular immune response | |  | | | | | | | | |
|  | *tcrα* | 0.85 | -0.23 | 0.4 | 1.82 | **0.86** | 0.017 | 0.8 | -0.32 | 0.56 |
|  | *ighm* | 1.35 | 0.43 | 0.3 | 1.92 | **0.94** | 0.027 | 0.99 | -0.009 | 0.99 |
|  | *ilc* | 1.27 | 0.34 | 0.34 | 1.7 | 0.78 | 0.11 | 0.68 | -0.55 | 0.39 |
| Nonspecific cytotoxic cell receptor | |  | | | | | | | | |
|  | *nccrp1* | 1.27 | 0.35 | 0.38 | 0.93 | -0.12 | 0.73 | 2.67 | **1.42** | 0.0004 |
| Proteolysis and apoptotic process | |  | | | | | | | | |
|  | *lgals1* | 1.1 | 0.14 | 0.6 | 0.96 | -0.06 | 0.78 | 2.65 | **1.4** | 0.012 |
|  | *prf1* | 0.52 | -0.93 | 0.66 | 7.4 | 2.9 | 0.08 | 5.19 | **2.38** | 0.004 |

**Supplementary Table S8.** Differentially expressed genes (DEGs) in intestine from non-vaccinated animals.

| Biological process | Gene | Intestine | | | | | | | | |
| --- | --- | --- | --- | --- | --- | --- | --- | --- | --- | --- |
|  |  |  |  |  |  |  |  |  |  |  |
|  |  | 24 h pi | | | 48 h pi | | | 72 h pi | | |
|  |  | FC | Log_2_FC | *p*-value | FC | Log_2_FC | *p*-value | FC | Log_2_FC | *p*-value |
| Viral recognition | | | | | | | | | | |
|  | *tlr5* | 0.9 | -0.15 | 0.67 | 2.28 | 1.19 | 0.54 | 4.5 | **2.17** | 0.01 |
| Regulation of innate and adaptive immune response | |  | | | | | | | | |
|  | *clec10a* | 0.41 | -1.29 | 0.25 | 1.47 | 0.56 | 0.25 | 4.92 | **2.3** | 0.002 |
|  | *tgfβ1* | 0.67 | **-0.58** | 0.02 | 0.5 | **-1** | 0.001 | 1.1 | 0.11 | 0.73 |
| Type I IFN response | |  | | | | | | | | |
|  | *pkr* | 0.99 | -0.02 | 0.91 | 0.74 | -0.43 | 0.053 | 1.5 | 0.57 | 0.18 |
|  | *isg15* | 1.92 | **0.94** | 0.04 | 0.62 | -0.69 | 0.27 | 1.96 | **0.97** | 0.04 |
|  | *mx1* | 2.61 | **1.38** | 0.03 | 1.16 | 0.21 | 0.69 | 3.74 | 1.9 | 0.09 |
|  | *mx2* | 2.68 | **1.42** | 0.03 | 0.77 | -0.38 | 0.34 | 2.1 | 1.06 | 0.14 |
|  | *mx3* | 2.16 | **1.11** | 0.03 | 1.6 | 0.67 | 0.31 | 1.79 | 0.84 | 0.16 |
|  | *ifi30* | 0.061 | **-4.04** | 0.004 | 0.033 | **-4.92** | 0.0006 | 0.13 | **-2.94** | 0.04 |
| Inflammatory process | |  | | | | | | | | |
|  | *il1β* | 0.61 | -0.71 | 0.08 | 0.5 | -0.91 | 0.07 | 5.3 | **2.41** | 0.004 |
|  | *il8* | 0.24 | **-2.06** | 0.001 | 0.54 | -0.88 | 0.11 | 1.14 | 0.19 | 0.68 |
|  | *il10* | 0.69 | -0.54 | 0.19 | 1.01 | 0.022 | 0.97 | 2.8 | **1.48** | 0.01 |
|  | *tnfα* | 1.04 | 0.053 | 0.84 | 1.3 | 0.37 | 0.71 | 1.97 | 0.98 | 0.16 |
|  | *ck3* | 0.33 | **-1.6** | 0.01 | 0.262 | **-1.93** | 0.0007 | 0.63 | -0.67 | 0.16 |
|  | *ccr3* | 0.67 | **-0.57** | 0.02 | 0.88 | -0.19 | 0.34 | 0.67 | -0.54 | 0.28 |
|  | *ck8* | 0.66 | -0.59 | 0.098 | 0.55 | **-0.86** | 0.04 | 0.89 | -0.16 | 0.8 |
|  | *ck10* | 0.28 | **-1.83** | 0.04 | 0.16 | **-2.64** | 0.001 | 0.46 | **-1.12** | 0.03 |
|  | *cox2* | 0.67 | -0.58 | 0.14 | 0.63 | -0.66 | 0.22 | 1.56 | 0.64 | 0.23 |
|  | *csf1r* | 1.12 | 0.17 | 0.41 | 1.07 | 0.1 | 0.74 | 1.95 | **0.96** | 0.03 |
|  | *ncf4* | 0.63 | -0.66 | 0.24 | 0.94 | -0.08 | 0.86 | 2.05 | **1.04** | 0.019 |
|  | *elam* | 0.7 | -0.52 | 0.12 | 0.83 | -0.26 | 0.5 | 1.1 | 0.14 | 0.74 |
| Antigen processing and presentation | |  | | | | | | | | |
|  | *iclp* | 0.87 | -0.21 | 0.5 | 0.67 | **-0.58** | 0.0005 | 0.92 | -0.11 | 0.63 |
|  | *mrc1* | 0.44 | **-1.18** | 0.01 | 0.47 | **-1.09** | 0.0008 | 0.79 | -0.34 | 0.29 |
| Humoral and cellular immune response | |  | | | | | | | | |
|  | *tcrα* | 0.95 | -0.067 | 0.84 | 0.63 | **-0.67** | 0.007 | 1.16 | 0.28 | 0.33 |
|  | *tcrβ* | 0.78 | -0.36 | 0.41 | 0.63 | **-0.67** | 0.012 | 1.12 | 0.16 | 0.53 |
|  | *ighm* | 0.46 | -1.13 | 0.21 | 0.5 | -0.98 | 0.18 | 0.38 | -1.39 | 0.07 |
|  | *ilc* | 0.83 | -0.27 | 0.28 | 1.33 | 0.42 | 0.47 | 2.43 | **1.28** | 0.02 |
|  | *cd276* | 0.38 | **-1.4** | 0.004 | 0.6 | **-0.74** | 0.04 | 1.34 | 0.42 | 0.36 |
| Nonspecific cytotoxic cell receptor | |  |  |  |  |  |  |  |  |  |
|  | *nccrp1* | 1.51 | **0.59** | 0.04 | 0.56 | **-0.84** | 0.004 | 1.0 | 0.01 | 0.95 |
| Proteolysis and apoptotic process | |  | | | | | | | | |
|  | *ctsb* | 0.56 | -0.83 | 0.056 | 0.32 | **-1.64** | 0.0004 | 0.91 | -0.13 | 0.76 |
|  | *lgals1* | 0.43 | **-1.22** | 0.01 | 0.3 | **-1.74** | 0.0003 | 0.4 | **-1.32** | 0.004 |
|  | *perp* | 0.96 | -0.065 | 0.68 | 0.54 | -0.89 | 0.17 | 1.37 | 0.46 | 0.18 |
|  | *prf1* | 1.71 | 0.77 | 0.55 | 0.09 | **-3.47** | 0.0004 | 0.23 | **-2.12** | 0.014 |
| Molecular stress response | |  | | | | | | | | |
|  | *hsp70* | 1.31 | 0.39 | 0.1 | 0.91 | -0.13 | 0.5 | 1.61 | **0.69** | 0.009 |
|  | *hsp90* | 1.11 | 0.15 | 0.45 | 0.5 | **-1** | 0.001 | 1.06 | 0.09 | 0.61 |

**Supplementary Table S9.** Differentially expressed genes (DEGs) in intestine from vaccinated animals.

| Biological process | Gene | Intestine | | | | | | | | |
| --- | --- | --- | --- | --- | --- | --- | --- | --- | --- | --- |
|  |  |  |  |  |  |  |  |  |  |  |
|  |  | 24 h pi | | | 48 h pi | | | 72 h pi | | |
|  |  | FC | Log_2_FC | *p*-value | FC | Log_2_FC | *p*-value | FC | Log_2_FC | *p*-value |
| Viral recognition | |  | | | | | | | | |
|  | *tlr5* | 1.14 | 0.19 | 0.64 | 0.89 | -0.15 | 0.84 | 1.54 | 0.62 | 0.16 |
| Regulation of innate and adaptive immune response | |  |  |  |  |  |  |  |  |  |
|  | *clec10a* | 0.34 | -1.54 | 0.16 | 0.76 | -0.25 | 0.19 | 2.04 | 1.03 | 0.18 |
|  | *tgfβ1* | 0.77 | -0.37 | 0.18 | 0.43 | **-1.23** | 0.0005 | 0.89 | -0.16 | 0.4 |
| Type I IFN response | |  | | | | | | | | |
|  | *pkr* | 1.12 | 0.16 | 0.41 | 0.84 | -0.25 | 0.19 | 1.06 | 0.08 | 0.72 |
|  | *isg15* | 1.72 | **0.78** | 0.023 | 0.72 | -0.48 | 0.37 | 1.3 | 0.4 | 0.37 |
|  | *mx1* | 3.66 | **1.87** | 0.045 | 1.61 | 0.68 | 0.3 | 2.7 | 1.44 | 0.18 |
|  | *mx2* | 1.87 | 0.9 | 0.17 | 0.75 | -0.42 | 0.58 | 1.2 | 0.24 | 0.76 |
|  | *mx3* | 2.7 | 1.43 | 0.057 | 1.18 | 0.23 | 0.7 | 1.3 | 0.41 | 0.51 |
|  | *ifi30* | 0.08 | **-3.59** | 0.007 | 0.02 | **-5.44** | 0.000005 | 0.113 | **-3.14** | 0.035 |
| Inflammatory process | |  | | | | | | | | |
|  | *il1β* | 0.65 | -0.62 | 0.08 | 0.64 | -0.64 | 0.08 | 2.088 | **1.06** | 0.022 |
|  | *il8* | 0.39 | **-1.34** | 0.006 | 0.37 | **-1.44** | 0.011 | 0.425 | **-1.23** | 0.046 |
|  | *il10* | 0.93 | -0.1 | 0.77 | 0.61 | -0.7 | 0.15 | 1.28 | 0.36 | 0.37 |
|  | *tnfα* | 0.78 | -0.35 | 0.12 | 0.76 | -0.39 | 0.31 | 1.2 | 0.26 | 0.5 |
|  | *ck3* | 0.41 | **-1.27** | 0.03 | 0.18 | **-2.46** | 0.0002 | 0.39 | **-1.35** | 0.02 |
|  | *ccr3* | 0.76 | -0.39 | 0.21 | 0.55 | **-0.86** | 0.004 | 0.72 | -0.46 | 0.015 |
|  | *ck8* | 1.23 | 0.29 | 0.43 | 0.76 | -0.4 | 0.19 | 0.66 | -0.59 | 0.39 |
|  | *ck10* | 0.53 | -0.91 | 0.29 | 0.17 | **-2.53** | 0.00004 | 0.57 | -0.81 | 0.09 |
|  | *cox2* | 0.69 | -0.53 | 0.2 | 0.49 | **-1.02** | 0.018 | 0.87 | -0.19 | 0.6 |
|  | *csf1r* | 1.5 | **0.58** | 0.013 | 1.5 | 0.57 | 0.051 | 1.37 | 0.46 | 0.32 |
|  | *ncf4* | 0.69 | -0.53 | 0.34 | 0.57 | -0.8 | 0.12 | 0.9 | -0.15 | 0.66 |
|  | *elam* | 1.0 | 0.002 | 0.99 | 0.72 | -0.47 | 0.18 | 0.73 | -0.45 | 0.15 |
| Antigen processing and presentation | |  | | | | | | | | |
|  | *iclp* | 0.99 | -0.012 | 0.97 | 0.91 | -0.13 | 0.36 | 0.83 | -0.27 | 0.29 |
|  | *mrc1* | 0.69 | -0.53 | 0.13 | 0.33 | **-1.617** | 0.0002 | 0.68 | -0.56 | 0.14 |
| Humoral and cellular immune response | |  | | | | | | | | |
|  | *tcrα* | 1.05 | 0.07 | 0.88 | 0.9 | -0.15 | 0.4 | 0.74 | -0.43 | 0.23 |
|  | *tcrβ* | 1.12 | 0.16 | 0.7 | 1.21 | 0.27 | 0.24 | 0.91 | -0.14 | 0.66 |
|  | *ighm* | 0.81 | -0.3 | 0.59 | 0.19 | **-2.39** | 0.007 | 0.54 | -0.88 | 0.13 |
|  | *ilc* | 0.95 | -0.06 | 0.85 | 0.86 | -0.21 | 0.66 | 1.34 | 0.43 | 0.18 |
|  | *cd276* | 0.73 | -0.45 | 0.12 | 0.35 | **-1.51** | 0.00006 | 0.95 | -0.07 | 0.83 |
| Nonspecific cytotoxic cell receptor | |  |  |  |  |  |  |  |  |  |
|  | *nccrp1* | 1.49 | **0.58** | 0.045 | 1.77 | **0.82** | 0.0007 | 0.87 | -0.2 | 0.53 |
| Proteolysis and apoptotic process | |  | | | | | | | | |
|  | *ctsb* | 0.58 | -0.77 | 0.074 | 0.34 | **-1.53** | 0.0001 | 0.67 | -0.58 | 0.18 |
|  | *lgals1* | 0.88 | -0.18 | 0.59 | 0.25 | **-2** | 0.0002 | 0.57 | -0.82 | 0.055 |
|  | *perp* | 0.73 | -0.45 | 0.18 | 0.57 | **-0.81** | 0.0009 | 0.6 | -0.73 | 0.18 |
|  | *prf1* | 3.03 | 1.6 | 0.13 | 0.28 | **-1.84** | 0.048 | 0.95 | -0.07 | 0.92 |
| Molecular stress response | |  | | | | | | | | |
|  | *hsp70* | 1.08 | 0.12 | 0.63 | 1.16 | 0.22 | 0.11 | 1.24 | 0.31 | 0.15 |
|  | *hsp90* | 1.02 | 0.027 | 0.9 | 0.69 | **-0.52** | 0.032 | 0.84 | -0.24 | 0.16 |

**Supplementary Table S10.** Differentially expressed genes (DEGs) in intestine from mock-vaccinated animals.

| Biological process | Gene | Intestine | | | | | | | | |
| --- | --- | --- | --- | --- | --- | --- | --- | --- | --- | --- |
|  |  |  |  |  |  |  |  |  |  |  |
|  |  | 24 h pi | | | 48 h pi | | | 72 h pi | | |
|  |  | FC | Log_2_FC | *p*-value | FC | Log_2_FC | *p*-value | FC | Log_2_FC | *p*-value |
| Viral recognition | |  | | | | | | | | |
|  | *tlr5* | 0.65 | -0.63 | 0.08 | 0.64 | -0.63 | 0.44 | 0.99 | -0.002 | 0.99 |
| Regulation of innate and adaptive immune response | |  |  |  |  |  |  |  |  |  |
|  | *clec10a* | 0.22 | -2.14 | 0.07 | 0.78 | -0.35 | 0.15 | 1.45 | 0.54 | 0.62 |
|  | *tgfβ1* | 0.79 | -0.33 | 0.19 | 0.65 | **-0.62** | 0.03 | 0.77 | -0.37 | 0.18 |
| Type I IFN response | |  | | | | | | | | |
|  | *pkr* | 1.22 | 0.29 | 0.18 | 0.68 | **-0.55** | 0.035 | 0.97 | -0.04 | 0.88 |
|  | *isg15* | 1.52 | 0.6 | 0.25 | 0.63 | -0.66 | 0.088 | 1.28 | 0.35 | 0.5 |
|  | *mx1* | 0.94 | -0.09 | 0.88 | 0.67 | -0.58 | 0.14 | 1.2 | 0.26 | 0.78 |
|  | *mx2* | 1.23 | 0.3 | 0.58 | 0.33 | **-1.59** | 0.004 | 0.99 | -0.02 | 0.98 |
|  | *mx3* | 1.18 | 0.24 | 0.68 | 0.51 | **-0.98** | 0.04 | 0.81 | -0.29 | 0.66 |
|  | *ifi30* | 1.57 | 0.65 | 0.48 | 0.22 | -2.21 | 0.09 | 0.89 | -0.16 | 0.92 |
| Inflammatory process | |  | | | | | | | | |
|  | *il1β* | 0.99 | -0.01 | 0.08 | 1.37 | 0.45 | 0.07 | 1.02 | 0.025 | 0.08 |
|  | *il8* | 0.63 | -0.66 | 0.17 | 0.49 | **-1.02** | 0.03 | 0.37 | **-1.45** | 0.02 |
|  | *il10* | 0.84 | -0.25 | 0.51 | 0.65 | -0.61 | 0.23 | 0.98 | -0.035 | 0.9 |
|  | *tnfα* | 0.69 | **-0.54** | 0.006 | 0.7 | -0.52 | 0.17 | 0.73 | -0.45 | 0.17 |
|  | *ck3* | 0.88 | -0.18 | 0.69 | 0.42 | **-1.26** | 0.009 | 0.44 | -1.17 | 0.09 |
|  | *ccr3* | 0.84 | -0.26 | 0.3 | 0.83 | -0.27 | 0.36 | 0.64 | **-0.64** | 0.03 |
|  | *ck8* | 1.43 | 0.51 | 0.13 | 1.02 | 0.023 | 0.94 | 0.85 | -0.23 | 0.75 |
|  | *ck10* | 0.92 | -0.12 | 0.87 | 0.46 | **-1.11** | 0.03 | 0.98 | -0.03 | 0.96 |
|  | *cox2* | 0.74 | -0.44 | 0.32 | 0.88 | -0.17 | 0.65 | 0.9 | -0.15 | 0.64 |
|  | *csf1r* | 1.11 | 0.14 | 0.52 | 0.99 | -0.01 | 0.96 | 0.9 | -0.14 | 0.66 |
|  | *ncf4* | 0.82 | -0.27 | 0.59 | 0.75 | -0.42 | 0.24 | 0.7 | -0.52 | 0.27 |
|  | *elam* | 0.66 | **-0.6** | 0.045 | 0.96 | -0.06 | 0.86 | 0.86 | -0.22 | 0.5 |
| Antigen processing and presentation | |  | | | | | | | | |
|  | *iclp* | 1.12 | 0.17 | 0.62 | 0.88 | -0.18 | 0.26 | 1.01 | 0.02 | 0.93 |
|  | *mrc1* | 0.93 | -0.1 | 0.76 | 0.57 | **-0.82** | 0.02 | 0.69 | -0.52 | 0.087 |
| Humoral and cellular immune response | |  | | | | | | | | |
|  | *tcrα* | 1.19 | 0.25 | 0.51 | 1.02 | 0.02 | 0.91 | 0.6 | -0.73 | 0.076 |
|  | *tcrβ* | 1.36 | 0.44 | 0.3 | 0.99 | -0.01 | 0.95 | 0.77 | -0.36 | 0.2 |
|  | *ighm* | 1.44 | 0.53 | 0.3 | 0.75 | -0.42 | 0.48 | 0.51 | **-0.96** | 0.011 |
|  | *ilc* | 1.25 | 0.32 | 0.44 | 1.03 | 0.05 | 0.93 | 0.99 | -0.002 | 0.99 |
|  | *cd276* | 0.79 | -0.34 | 0.33 | 0.63 | **-0.66** | 0.008 | 1.04 | 0.009 | 0.97 |
| Nonspecific cytotoxic cell receptor | |  | | | | | | | | |
|  | *nccrp1* | 1.3 | 0.38 | 0.11 | 1.03 | 0.04 | 0.87 | 0.74 | -0.42 | 0.14 |
| Proteolysis and apoptotic process | |  |  |  |  |  |  |  |  |  |
|  | *ctsb* | 0.84 | -0.24 | 0.59 | 0.52 | **-0.93** | 0.03 | 0.71 | -0.5 | 0.31 |
|  | *lgals1* | 0.83 | -0.27 | 0.4 | 0.53 | **-0.9** | 0.01 | 0.52 | **-0.93** | 0.044 |
|  | *perp* | 1.62 | **0.7** | 0.001 | 0.78 | -0.36 | 0.18 | 1.03 | 0.039 | 0.93 |
|  | *prf1* | 3.4 | 1.77 | 0.69 | 0.51 | -0.97 | 0.26 | 0.96 | -0.063 | 0.93 |
| Molecular stress response | |  | | | | | | | | |
|  | *hsp70* | 0.87 | -0.19 | 0.3 | 0.97 | -0.05 | 0.79 | 0.96 | -0.06 | 0.82 |
|  | *hsp90* | 0.94 | -0.095 | 0.6 | 0.71 | **-0.49** | 0.001 | 0.74 | -0.44 | 0.75 |

**Supplementary Table S11.** Differentially expressed genes (DEGs) in caudal fin from non-vaccinated animals.

| Biological process | Gene | Caudal fin | | | | | | | | |
| --- | --- | --- | --- | --- | --- | --- | --- | --- | --- | --- |
|  |  |  |  |  |  |  |  |  |  |  |
|  |  | 24 h pi | | | 48 h pi | | | 72 h pi | | |
|  |  | FC | Log_2_FC | *p*-value | FC | Log_2_FC | *p*-value | FC | Log_2_FC | *p*-value |
| Viral recognition | | | | | | | | | | |
|  | *tlr5* | 0.88 | -0.19 | 0.4 | 0.7 | -0.48 | 0.28 | 0.58 | **-0.79** | 0.028 |
| Regulation of innate and adaptive immune response | |  | | | | | | | | |
|  | *tgfβ1* | 1.05 | 0.07 | 0.77 | 0.56 | **-0.83** | 0.007 | 0.76 | -0.4 | 0.25 |
| Type I IFN response | |  | | | | | | | | |
|  | *irf1* | 1.06 | 0.08 | 0.81 | 0.64 | -0.63 | 0.15 | 0.68 | -0.55 | 0.19 |
|  | *pkr* | 1.42 | **0.50** | 0.008 | 0.99 | -0.01 | 0.96 | 0.82 | -0.29 | 0.4 |
|  | *isg15* | 1.1 | 0.14 | 0.83 | 0.76 | -0.4 | 0.39 | 1.69 | 0.75 | 0.056 |
|  | *mx1* | 1.3 | 0.39 | 0.35 | 0.77 | -0.37 | 0.29 | 0.96 | -0.06 | 0.88 |
|  | *mx2* | 2.27 | 1.18 | 0.12 | 0.66 | -0.59 | 0.29 | 0.93 | -0.1 | 0.85 |
|  | *mx3* | 1.28 | 0.36 | 0.45 | 1.19 | 0.26 | 0.65 | 1.18 | 0.24 | 0.58 |
|  | *ifi30* | 0.96 | -0.06 | 0.8 | 0.5 | **-1.01** | 0.019 | 0.42 | **-1.26** | 0.014 |
| Inflammatory process | |  | | | | | | | | |
|  | *il1β* | 1.93 | **0.95** | 0.0211 | 0.96 | -0.05 | 0.08 | 0.6 | -0.72 | 0.08 |
|  | *il8* | 0.93 | -0.1 | 0.82 | 1.44 | 0.52 | 0.26 | 0.54 | -0.9 | 0.07 |
|  | *ck3* | 1.61 | 0.69 | 0.12 | 0.82 | -0.29 | 0.45 | 0.45 | **-1.14** | 0.007 |
|  | *ccr3* | 1.28 | 0.35 | 0.14 | 0.42 | **-1.25** | 0.004 | 0.54 | **-0.88** | 0.041 |
|  | *ck10* | 1.7 | 0.76 | 0.13 | 0.45 | **-1.15** | 0.024 | 0.27 | **-1.88** | 0.015 |
|  | *tnfα* | 0.78 | -0.36 | 0.29 | 0.91 | -0.13 | 0.57 | 0.99 | -0.01 | 0.97 |
|  | *cox2* | 1.02 | 0.02 | 0.91 | 0.86 | -0.22 | 0.44 | 0.76 | -0.4 | 0.4 |
|  | *csf1r* | 0.73 | -0.46 | 0.07 | 0.71 | **-0.5** | 0.049 | 0.96 | -0.05 | 0.88 |
|  | *elam* | 0.74 | -0.44 | 0.15 | 0.83 | -0.27 | 0.39 | 0.82 | -0.29 | 0.58 |
| Antigen processing and presentation | |  | | | | | | | | |
|  | *mhcIIα* | 0.82 | -0.29 | 0.34 | 0.43 | **-1.2** | 0.0004 | 0.45 | **-1.16** | 0.448 |
|  | *iclp* | 0.88 | -0.18 | 0.58 | 0.45 | **-1.15** | 0.003 | 0.45 | **-1.16** | 0.0008 |
|  | *mrc1* | 0.99 | -0.007 | 0.98 | 0.61 | **-0.71** | 0.025 | 0.93 | -0.1 | 0.58 |
| Humoral and cellular immune response | |  | | | | | | | | |
|  | *tcrα* | 0.85 | -0.23 | 0.39 | 0.54 | **-0.88** | 0.044 | 0.71 | -0.5 | 0.22 |
|  | *tcrβ* | 0.54 | **-0.9** | 0.0289 | 0.33 | **-1.6** | 0.0007 | 0.61 | -0.72 | 0.08 |
|  | *ighm* | 0.52 | -0.93 | 0.29 | 0.32 | **-1.63** | 0.032 | 0.57 | -0.82 | 0.18 |
|  | *ilc* | 0.54 | -0.88 | 0.14 | 0.31 | **-1.69** | 0.0085 | 0.51 | -0.97 | 0.054 |
|  | *cd48* | 1.46 | **0.54** | 0.0297 | 1.09 | 0.12 | 0.81 | 0.67 | -0.57 | 0.25 |
| Nonspecific cytotoxic cell receptor | |  | | | | | | | | |
|  | |  | | | | | | | | |
|  | *nccrp1* | 2.13 | **1.09** | 0.008 | 0.85 | -0.24 | 0.61 | 0.64 | -0.64 | 0.06 |
| Proteolysis and apoptotic process | |  | | | | | | | | |
|  | *casp1* | 1.48 | **0.56** | 0.0136 | 1.18 | 0.24 | 0.34 | 0.91 | -0.13 | 0.69 |
|  | *lgals1* | 1.1 | 0.12 | 0.87 | 0.35 | **-1.51** | 0.0018 | 0.67 | -0.58 | 0.31 |
|  | *perp* | 1.63 | **0.7** | 0.001 | 1.37 | 0.46 | 0.12 | 1.02 | 0.03 | 0.92 |
| Molecular stress response | |  | | | | | | | | |
|  | *hsp70* | 1.4 | 0.48 | 0.02 | 1.04 | 0.06 | 0.72 | 1.2 | 0.26 | 0.32 |
|  | *hsp90* | 1.1 | 0.1 | 0.69 | 0.71 | **-0.49** | 0.0285 | 1.1 | 0.14 | 0.64 |

**Supplementary Table S12.** Differentially expressed genes (DEGs) in caudal fin from vaccinated animals.

| Biological process | Gene | Caudal fin | | | | | | | | |
| --- | --- | --- | --- | --- | --- | --- | --- | --- | --- | --- |
|  |  |  |  |  |  |  |  |  |  |  |
|  |  | 24 h pi | | | 48 h pi | | | 72 h pi | | |
|  |  | FC | Log_2_FC | *p*-value | FC | Log_2_FC | *p*-value | FC | Log_2_FC | *p*-value |
| Viral recognition | |  | | | | | | | | |
|  | *tlr5* | 0.44 | -1.17 | 0.06 | 0.68 | -0.56 | 0.22 | 1.5 | 0.58 | 0.16 |
| Regulation of innate and adaptive immune response | |  |  |  |  |  |  |  |  |  |
|  | *tgfβ1* | 1.02 | 0.04 | 0.9 | 0.93 | -0.11 | 0.66 | 0.99 | -0.02 | 0.96 |
| Type I IFN response | |  |  |  |  |  |  |  |  |  |
|  | *irf1* | 0.75 | -0.41 | 0.35 | 0.57 | **-0.81** | 0.044 | 0.92 | -0.12 | 0.76 |
|  | *pkr* | 0.87 | -0.2 | 0.53 | 1.1 | 0.11 | 0.62 | 1.64 | 0.72 | 0.08 |
|  | *isg15* | 1.35 | 0.44 | 0.51 | 0.55 | -0.86 | 0.26 | 2.42 | **1.27** | 0.025 |
|  | *mx1* | 1.51 | 0.59 | 0.37 | 1.01 | 0.01 | 0.98 | 2.09 | **1.06** | 0.018 |
|  | *mx2* | 1.7 | 0.77 | 0.43 | 0.61 | -0.72 | 0.35 | 2.05 | **1.03** | 0.033 |
|  | *mx3* | 0.97 | -0.04 | 0.95 | 0.87 | -0.19 | 0.8 | 3.54 | **2.28** | 0.002 |
|  | *ifi30* | 0.99 | -0.01 | 0.96 | 0.81 | -0.3 | 0.37 | 0.88 | -0.18 | 0.67 |
| Inflammatory process | |  | | | | | | | | |
|  | *il1β* | 1.72 | 0.78 | 0.51 | 0.9 | -0.16 | 0.08 | 0.47 | -1.09 | 0.08 |
|  | *il8* | 0.63 | -0.68 | *0.39* | 0.94 | -0.08 | 0.84 | 0.48 | **-1.08** | 0.042 |
|  | *ck3* | 0.98 | -0.035 | 0.94 | 0.87 | -0.19 | 0.57 | 0.85 | -0.23 | 0.59 |
|  | *ccr3* | 1.44 | 0.53 | 0.057 | 0.82 | -0.29 | 0.37 | 0.84 | -0.25 | 0.52 |
|  | *ck10* | 1.44 | 0.52 | 0.26 | 0.56 | -0.84 | 0.1 | 0.4 | -1.34 | 0.057 |
|  | *tnfα* | 0.54 | **-0.89** | 0.013 | 0.74 | -0.43 | 0.29 | 1.24 | 0.31 | 0.4 |
|  | *cox2* | 0.59 | **-0.77** | 0.01 | 0.97 | -0.04 | 0.86 | 1.03 | 0.045 | 0.92 |
|  | *csf1r* | 0.63 | **-0.66** | 0.13 | 0.84 | -0.25 | 0.23 | 1.14 | 0.19 | 0.6 |
|  | *elam* | 0.51 | **-0.96** | 0.023 | 1.15 | 0.21 | 0.55 | 0.79 | -0.34 | 0.53 |
| Antigen processing and presentation | |  | | | | | | | | |
|  | *mhcIIα* | 0.88 | -0.18 | 0.47 | 0.6 | **-0.73** | 0.008 | 0.78 | -0.35 | 0.055 |
|  | *iclp* | 0.97 | -0.04 | 0.88 | 0.7 | -0.52 | 0.09 | 0.82 | -0.29 | 0.21 |
|  | *mrc1* | 0.97 | -0.04 | 0.9 | 0.96 | -0.06 | 0.84 | 1.45 | 0.53 | 0.2 |
| Humoral and cellular immune response | |  | | | | | | | | |
|  | *tcrα* | 0.63 | **-0.67** | 0.0163 | 0.64 | -0.64 | 0.13 | 0.89 | -0.16 | 0.68 |
|  | *tcrβ* | 0.65 | **-0.62** | 0.019 | 0.53 | **-0.92** | 0.011 | 1.05 | 0.065 | 0.87 |
|  | *ighm* | 0.95 | -0.07 | 0.9 | 0.57 | -0.81 | 0.098 | 0.83 | -0.27 | 0.58 |
|  | *ilc* | 0.64 | -0.64 | 0.2 | 0.43 | **-1.21** | 0.004 | 0.59 | -0.75 | 0.055 |
|  | *cd48* | 0.95 | -0.07 | 0.9 | 1.14 | 0.2 | 0.55 | 1.04 | 0.05 | 0.91 |
| Nonspecific cytotoxic cell receptor | |  |  |  |  |  |  |  |  |  |
|  | *nccrp1* | 1.94 | 0.96 | 0.06 | 1.2 | 0.3 | 0.23 | 1.4 | 0.48 | 0.17 |
| Proteolysis and apoptotic process | |  | | | | | | | | |
|  | *casp1* | 1.26 | 0.33 | 0.25 | 1.56 | **0.64** | 0.038 | 1.54 | 0.62 | 0.11 |
|  | *lgals1* | 1.7 | 0.76 | 0.35 | 0.69 | -0.53 | 0.24 | 0.83 | -0.26 | 0.66 |
|  | *perp* | 1.24 | 0.31 | 0.27 | 1.12 | 0.16 | 0.53 | 1.55 | 0.63 | 0.11 |
| Molecular stress response | |  |  |  |  |  |  |  |  |  |
|  | *hsp70* | 1.03 | 0.04 | 0.86 | 1.1 | 0.12 | 0.53 | 1.5 | 0.6 | 0.07 |
|  | *hsp90* | 0.78 | -0.36 | 0.25 | 0.88 | -0.18 | 0.34 | 1.27 | 0.34 | 0.3 |

**Supplementary Table S13.** Differentially expressed genes (DEGs) in caudal fin from mock-vaccinated animals.

| Biological process | Gene | Caudal fin | | | | | | | | |
| --- | --- | --- | --- | --- | --- | --- | --- | --- | --- | --- |
|  |  |  |  |  |  |  |  |  |  |  |
|  |  | 24 h pi | | | 48 h pi | | | 72 h pi | | |
|  |  | FC | Log_2_FC | *p*-value | FC | Log_2_FC | *p*-value | FC | Log_2_FC | *p*-value |
| Viral recognition | | | | | | | | | | |
|  | *tlr5* | 0.64 | **-0.64** | 0.007 | 1.07 | 0.1 | 0.83 | 0.99 | -0.008 | 0.99 |
| Regulation of innate and adaptive immune response | |  |  |  |  |  |  |  |  |  |
|  | *tgfβ1* | 1.12 | 0.16 | 0.42 | 0.97 | -0.05 | 0.88 | 0.98 | -0.03 | 0.94 |
| Type I IFN response | |  | | | | | | | | |
|  | *irf1* | 1.01 | 0.01 | 0.97 | 0.71 | -0.5 | 0.18 | 0.82 | -0.29 | 0.54 |
|  | *pkr* | 1.27 | 0.35 | 0.25 | 1.2 | 0.27 | 0.34 | 1.08 | 0.11 | 0.74 |
|  | *isg15* | 1.87 | 0.9 | 0.25 | 1.75 | **0.81** | 0.028 | 2.08 | 1.05 | 0.17 |
|  | *mx1* | 1.54 | 0.62 | 0.23 | 0.86 | -0.22 | 0.48 | 1.68 | 0.75 | 0.16 |
|  | *mx2* | 1.52 | 0.6 | 0.43 | 0.66 | -0.61 | 0.21 | 2.0 | 0.99 | 0.095 |
|  | *mx3* | 1.27 | 0.34 | 0.48 | 1.0 | 0.0 | 0.99 | 1.3 | 0.38 | 0.46 |
|  | *ifi30* | 1.28 | 0.36 | 0.05 | 0.48 | **-1.06** | 0.015 | 0.71 | -0.48 | 0.29 |
| Inflammatory process | |  | | | | | | | | |
|  | *il1β* | 0.89 | -0.17 | 0.68 | 1.4 | 0.5 | 0.08 | 0.58 | -0.77 | 0.08 |
|  | *il8* | 0.51 | -0.98 | 0.13 | 1.96 | **0.97** | 0.004 | 0.78 | -0.36 | 0.5 |
|  | *ck3* | 1.07 | 0.1 | 0.82 | 0.58 | -0.79 | 0.072 | 0.66 | -0.6 | 0.19 |
|  | *ccr3* | 1.52 | **0.6** | 0.017 | 0.33 | **-1.58** | 0.0007 | 0.92 | -0.12 | 0.76 |
|  | *ck10* | 2.03 | **1.02** | 0.04 | 0.38 | **-1.41** | 0.016 | 0.57 | -0.81 | 0.29 |
|  | *tnfα* | 0.68 | -0.55 | 0.18 | 1.4 | 0.5 | 0.16 | 1.24 | 0.31 | 0.47 |
|  | *cox2* | 0.56 | **-0.84** | 0.004 | 1.21 | 0.27 | 0.36 | 0.82 | -0.28 | 0.55 |
|  | *csf1r* | 0.76 | -0.39 | 0.03 | 0.95 | -0.08 | 0.64 | 1.04 | 0.06 | 0.89 |
|  | *elam* | 0.56 | **-0.83** | 0.035 | 1.02 | 0.03 | 0.93 | 0.63 | -0.66 | 0.26 |
| Antigen processing and presentation | |  | | | | | | | | |
|  | *mhcIIα* | 0.98 | -0.02 | 0.94 | 0.38 | **-1.38** | 0.001 | 0.72 | -0.47 | 0.13 |
|  | *iclp* | 1.2 | 0.26 | 0.36 | 0.48 | **-1.07** | 0.005 | 0.73 | -0.45 | 0.13 |
|  | *mrc1* | 1.24 | 0.3 | 0.27 | 0.7 | -0.51 | 0.77 | 1.03 | 0.05 | 0.91 |
| Humoral and cellular immune response | |  | | | | | | | | |
|  | *tcrα* | 0.94 | -0.096 | 0.66 | 1.13 | 0.17 | 0.66 | 0.76 | -0.39 | 0.33 |
|  | *tcrβ* | 0.91 | -0.14 | 0.6 | 0.6 | -0.74 | 0.087 | 0.78 | -0.35 | 0.36 |
|  | *ighm* | 1.85 | 0.89 | 0.15 | 0.63 | -0.67 | 0.22 | 0.83 | -0.27 | 0.63 |
|  | *ilc* | 1.2 | 0.25 | 0.58 | 0.66 | -0.59 | 0.18 | 0.53 | **-0.91** | 0.029 |
|  | *cd48* | 1.36 | 0.44 | 0.22 | 1.03 | 0.045 | 0.89 | 1.21 | 0.27 | 0.64 |
| Nonspecific cytotoxic cell receptor | |  |  |  |  |  |  |  |  |  |
|  | *nccrp1* | 1.93 | 0.95 | 0.088 | 0.95 | -0.07 | 0.81 | 1.12 | 0.15 | 0.71 |
| Proteolysis and apoptotic process | |  |  |  |  |  |  |  |  |  |
|  | *casp1* | 1.28 | 0.36 | 0.11 | 1.32 | 0.41 | 0.17 | 1.12 | 0.16 | 0.66 |
|  | *lgals1* | 1.66 | 0.73 | 0.39 | 0.97 | -0.037 | 0.89 | 0.59 | -0.76 | 0.2 |
|  | *perp* | 1.2 | 0.23 | 0.37 | 0.96 | -0.06 | 0.8 | 1.13 | 0.18 | 0.61 |
| Molecular stress response | |  | | | | | | | | |
|  | *hsp70* | 1.15 | 0.2 | 0.38 | 1.44 | **0.53** | 0.016 | 1.17 | 0.23 | 0.41 |
|  | *hsp90* | 0.82 | -0.28 | 0.31 | 0.91 | -0.13 | 0.52 | 1.0 | 0.006 | 0.99 |
